# Tumor-dependent increase of serum amino acid levels in breast cancer patients has diagnostic potential and correlates with molecular tumor subtypes

**DOI:** 10.1186/1479-5876-11-290

**Published:** 2013-11-16

**Authors:** Isabel Poschke, Yumeng Mao, Rolf Kiessling, Jana de Boniface

**Affiliations:** 1 Department of Oncology and Pathology, Cancer Center Karolinska, Karolinska Institutet, Stockholm, Sweden; 2 German Cancer Research Center, DKFZ, Heidelberg, Germany; 3 Department of Molecular Medicine and Surgery, Karolinska Institutet, Stockholm, Sweden; 4 Department of Breast and Endocrine Surgery, Karolinska University Hospital, Stockholm, Sweden

**Keywords:** Breast cancer, Serum amino acids, Molecular subtypes, Tumor immunology

## Abstract

**Background:**

Malignancies induce changes in the levels of serum amino acids (AA), which may offer diagnostic potential. Furthermore, changes in AA levels are associated with immune cell function. In this study, serum AA levels were studied in breast cancer patients *versus* patients with benign breast lesions.

**Methods:**

In a prospective study, serum levels of 15 AA were measured by high performance liquid chromatography before and after surgery in 41 breast cancer patients (BrCA) and nine patients with benign breast lesions (healthy donors, HD). Results were analyzed in relation to clinical tumor data and tested against immunological flow cytometry data. Principal component analysis was performed and the accuracy of AA levels as a potential diagnostic tool was tested.

**Results:**

Pre- but not postoperative serum AA levels were increased in BrCA in eight out of 15 AA compared with HD. Serum AA levels were highest in the most aggressive (basal-like) as compared with the least aggressive tumor subtype (luminal A). A principal component (PC1) of all measured AA correlated with a mainly pro-inflammatory immune profile, while a second one (PC2, selectively considering AA preoperatively differing between HD and BrCA) could predict health state with an area under the curve of 0.870.

**Conclusions:**

Breast cancer shows a tumor-dependent impact on serum AA levels, which varies with intrinsic tumor subtypes and is associated with a pro-inflammatory state. Serum AA levels need further evaluation as a potential diagnostic tool.

## Introduction

Amino acids (AA) are an integral part of most metabolomics analysis. As metabolomics measure downstream output rather than upstream gene and protein products, they offer the opportunity of an integrated evaluation of multiple pathways and their biological consequences [1]. Amino acid profiling has shown significant differences in AA levels between cancer patients and healthy controls [2-4], even though some of the reported findings remain to be confirmed. Furthermore, AA profiling has been shown to harbor potential as a diagnostic test, as lately reported by a Japanese group comparing plasma AA levels between lung cancer patients and healthy controls [5].

Building on the Warburg effect [6], the metabolic shift in tumor cells from respiration to fermentation should result in an increased demand for and consumption of amino acids, amongst other substances. Indeed, this has been shown by several publications [7,8]. However, an amino acid-rich substrate could inhibit tumor growth by cell cycle arrest and initiation of apoptosis in a murine model [9]. These contradictory findings may well represent a dose-dependent effect, rendering both the “depletion” and the “overload” model valid.

In addition to metabolite consumption by the tumor, it is also crucial to consider the role of amino acids for the pro- and anti-tumorigenic effect of immune cells. Activation of immune cells often leads to increases of baseline amino acid requirement and immune effector cells often rely on an external supply of certain amino acids. Lack of AA in this situation may affect the immune response negatively in several ways, including cell division, maturation, differentiation, migration and development of effector functions (reviewed in [10]).

We have previously shown that T lymphocytes in the blood, lymph nodes and tumor of the present breast cancer patient cohort expressed decreased levels of the TCR-zeta chain, which is essential for the transduction of stimulatory signals [11]. Others have shown that loss of zeta-chain expression can be a result of L-arginine (L-Arg) depletion [12,13]. Further, we observed an increased number of cells expressing the L-Arg-metabolizing enzyme arginase 1 in the breast cancer population compared with controls [14]. In addition to L-Arg, other amino acids such as tryptophan and the purine nucleoside adenosine have been associated with the suppressive activity of immune cells. Immunomodulation may also occur through products of amino acid catabolism, e.g. via the IDO-GCN2, arginase and mTOR pathways [15].

In the context of the immunological evaluation of patients with early-stage breast cancer [11,14,16] we measured the serum levels of 15 different amino acids by high performance liquid chromatography (HPLC) before and after surgery. Results were compared with serum samples from patients operated for benign breast lesions.

## Materials and methods

### Patients

This prospective study included patients with primary, unilateral, invasive breast cancer without preoperatively detected lymph node metastases. Sentinel lymph node biopsy (SLNB) was performed on all patients. Details on this patient population have previously been published [11] and are summarized in Table 1.

**Table 1:** Patient and tumor characteristics of 41 breast cancer patients

	
**Age (years) *median (range)***	62 (39–91)
**Tumor diameter (mm) *median (range)***	16 (6–100)
**Histological tumor type *N (%)***	
Ductal	35 (85.4)
Lobular	5 (12.2)
Medullar	1 (2.4)
**Estrogen receptor status *N (%)***	
Positive	33 (80.5)
Negative	8 (19.5)
**Progesterone receptor status *N (%)***	
Positive	28 (68.3)
Negative	13 (31.7)
**Her2-neu status (FISH**^**a**^**) *N (%)***	
Positive	3 (7.3)
Negative	38 (92.7)
**LVI**^**c **^ ***N (%)***	
Yes	10 (24.4)
No	31 (75.6)
**Elston histological grading *N (%)***	
1	8 (19.5)
2	19 (46.3)
3	14 (34.1)
**Ki 67 in tumor cells (%) *mean (sd)***	24.6 (19.9)
**Sentinel node result *N (%)***	
N0	29 (70.7)
N1	12 (29.3)
**Tumor intrinsic subtype *N (%)***	
Luminal A	18 (43.9)
Luminal B	14 (34.1)
Luminal B Her2-neu positive	1 (2.4)
Her2-neu over-expressing non-luminal	2 (4.9)
Basal-like	6 (14.6)

^*a*^*Fluorescence in vitro hybridization,*^*c*^*lymphovascular invasion; sd standard deviation.*

As controls, individuals scheduled for surgical removal of benign breast lesions, such as fibroadenomas or papillomas, were included.

All participants provided informed written consent before inclusion. This study was approved by the Regional Ethical Review Board at Karolinska Institutet, Stockholm.

### Surgical procedure and handling of specimens

All patients were operated under general anesthesia. Blood samples were taken before and 2–4 weeks after surgery. Peripheral blood mononuclear cells (PBMC) were isolated by density gradient centrifugation as described previously [11]. At each time point, 5 mL blood were collected in a tube without anti-coagulant. Blood was allowed to clot for 1h, then serum was collected and additionally purified by 15 minutes centrifugation at 1500 × g. After centrifugation the supernatant was aliquoted and stored at −20 degrees until further analysis.

### Tumor histopathological data

Data on tumor characteristics and lymph node status were extracted from each patient’s routine postoperative histopathological report. For the definition of intrinsic genomic subtypes, the surrogate parameters of estrogen and progesterone receptor positivity, Her2-neu status, and Ki67 labeling index were applied as described in the St Gallen consensus report from 2011 [17].

### High performance liquid chromatography (HPLC) and fluorescence detection

Amino acids in plasma samples were determined by gradient elution reversed-phase column liquid chromatography with fluorescence detection following precolumn derivatization with orthophtaldialdehyde/mercaptoethanol (OPA/MCE) reagent, following minor modification of the procedure described elsewhere [18]. Briefly, the HPLC system included a gradient pump Spectra Physics SP8800 (Spectra Physics, USA), a CMA/260 degasser (CMA Microdialysis), a CMA/280 Fluorescence detector (CMA Microdialysis) operating at excitation and emission wavelengths of 350 and 495 nm, respectively. The derivatization reagent was prepared as follows: 27 mg OPA were dissolved in 0.5 ml ethanol (99.5%). Thereafter, 5 ml of borate buffer (0.4 M boric acid adjusted to pH 10.4 with sodium hydroxide) were added followed by 20 μl of MCE. Typically, 10 μl samples were mixed with 10 μl of the OPA/MCE reagent by use of a CMA/200 Refrigerated Microsampler (CMA Microdialysis) equipped with a 20-μl loop and operating at +6°C. After 60 s reaction time, 10 μl volume was injected onto a HPLC column (60 × 4 mm i.d., Nucleosil 100 C18, 5μm; Knauer GmbH, Berlin, Germany). The mobile phase A was a 0.03 M sodium acetate buffer (pH 6.95) containing 2.5% (v/v) of methanol and 2% (v/v) of tetrahydrofuran and pumped at a flow rate of 1 ml/min. The amino acids were eluted by use of a linear gradient of methanol used as a mobile phase B and from 0 - 60% at 4 to 28 min. Thereafter, the column was regenerated with mobile phase A for 3 min. The chromatograms were recorded and integrated by use of a computerized data acquisition system (EZ Chrom data system, Scientific software Inc, CA, USA). The chemicals were purchased from Sigma Aldrich (St. Louis, MO, USA), methanol and tetrahydrofuran were from Merck (Darmstadt, Germany).

### Flow cytometric analysis of immunological parameters

Phenotype and functional properties of freshly isolated PBMC were analyzed by flow cytometry as previously described [11]. Additional file 1: Table S1 lists the antibody clones and staining conditions for the parameters described herein.

### Statistical methods

Prior to analysis, the distribution of all continuous variables was tested using the Shapiro-Wilk test. Based on data normality, parametric or non-parametric test options were chosen. Since median age and body mass index (BMI) of the control population were significantly lower than in breast cancer patients, all comparisons between the groups were adjusted for these two independent factors by additional linear regression. Any group differences are reported after adjustment throughout this paper.

Differences between pre- and postoperative amino acid levels were analyzed using the Wilcoxon signed rank test or the paired samples t-test, respectively, for healthy donors and breast cancer patients separately. The fold change from pre- to postoperative values was then compared between the two groups by linear regression, including adjustment for age and BMI.

**Figure 1:**
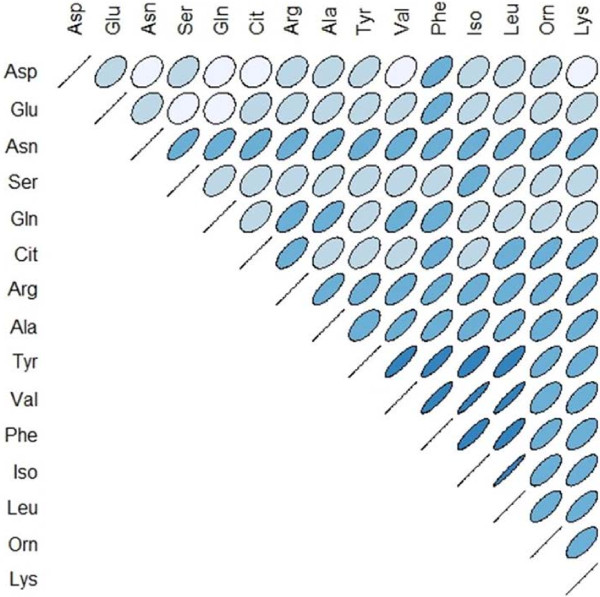
**Inter-correlation of preoperative serum amino acid levels.** Positive correlations are angled from lower left to upper right, negative correlations vice versa. The color indicates the R values from R = −1 (red) over light blue to R = 1 (dark blue). The R value is further illustrated by the roundness of the ellipse, with R = 0 a perfect circle. (n = 50, of which n = 41 breast cancer patients (BrCA) and n = 9 healthy donors (HD)).

Due to the extensive inter-correlation of amino acid levels (Figure 1), a principal component analysis (PCA) was performed after testing its adequacy by Kaiser-Meyer-Olkin measures and Bartlett’s test of sphericity.

For the analysis of covariation between the first principal component (PC1) and continuous immunological parameters, bivariate correlation was tested according to Pearson or Spearman’s rho, depending on data distribution.

A receiver operating characteristic (ROC) curve was generated based on a second principal component, including only those amino acids showing significant differences between healthy women and breast cancer patients. For the resulting PC2, its sensitivity and specificity to predict health state was analyzed, and the area under the curve (AUC) calculated.

When comparing continuous variables in more than two groups, e.g. in different tumor histological grades or intrinsic tumor subtypes, the Kruskal-Wallis test or ANOVA were performed.

IBM® SPSS® Statistics 21.0 software was used for all analyses and statistical significance was set at the 0.05 level for all tests.

## Results

The prospectively enrolled study population consisted of 44 patients with malignant and 11 patients with benign breast disease. For the analysis of serum amino acids, preoperative samples of 41 breast cancer patients and nine patients with benign breast lesions were available; pairs of pre- and postoperative serum values were obtained from 38 and nine patients, respectively. Data on patient and tumor characteristics are shown in Table 1.

### Generation of a principal component

The marked inter-correlation of most amino acids, with preoperative R values ranging from .339 to .974 (see Figure 1), suggested the use of dimension reduction which was performed using principal component analysis. Kaiser-Meyer-Olkin measures of sampling adequacy of .861 strengthened the adequacy of this procedure together with a highly significant Bartlett’s test of sphericity (p = 0.000). The extraction of only one factor (principal component 1, PC1), explaining 64.125% of the total variance, was suggested by an initial scree plot.

### Amino acid levels in preoperative serum are higher in cancer patients than in healthy controls

Healthy donors (HD) had a significantly lower median age (49 (34–63) *versus* 62 (39–91), p = 0.003) and body mass index (BMI; 21.7 (19–23) *versus* 24.1 (17–47), p = 0.022) than breast cancer patients (BrCA). Therefore, all subsequent comparisons between groups were controlled for age and BMI by linear regression.

Preoperative serum levels of eight out of 15 amino acids (Glu, Ser, Gln, Ala, Val, Phe, Ile, Leu) were significantly higher in breast cancer patients than healthy donors (see Table 2). The same difference was true for the PC1 (p = 0.014). This difference was absent in postoperative samples in all but 2 out of 15 amino acids (Glu and Val, see Table 3). Due to occasional high standard deviations, some rather high fold differences did not reach statistical significance.

**Table 2:** Amino acid concentrations in preoperative serum

**Amino acid**		**Serum concentration [μg/mL]**		**Fold difference**	***P-value***	**Trend**
		**BrCA [n = 41]**	**HD [n = 9]**			
**Aspartic acid**	Asp	**22.69 ± 8.4**	**15.14 ± 6.9**	1.50		
**Glutamic acid**	Glu	**159.98 ± 59.0**	**86.86 ± 27.4**	1.84	*0.021*	Higher in BrCA
**Asparagine**	Asn	**54.68 ± 14.8**	**47.96 ± 17.4**	1.14		
**Serine**	Ser	**168.34 ± 41.7**	**139.19 ± 45.2**	1.21	*0.032*	Higher in BrCA
**Glutamine**	Gln	**571.81 ± 174.3**	**393.82 ± 113.7**	1.45	*0.017*	Higher in BrCA
**Citrulline**	Cit	**41.20 ± 12.2**	**33.56 ± 8.5**	1.23		
**Arginine**	Arg	**162.63 ± 44.7**	**124.16 ± 47.6**	1.31		
**Alanine**	Ala	**571.50 ± 167.7**	**349.48 ± 119.7**	1.64	*0.005*	Higher in BrCA
**Tyrosine**	Tyr	**94.77 ± 31.3**	**63.18 ± 36.5**	1.50		
**Valine**	Val	**316.15 ± 68.1**	**205.66 ± 75.2**	1.54	*0.003*	Higher in BrCA
**Phenylalanine**	Phe	**91.51 ± 20.8**	**61.92 ± 22.2**	1.48	*0.015*	Higher in BrCA
**Isoleucine**	Ile	**93.77 ± 26.6**	**58.43 ± 24.6**	1.60	*0.011*	Higher in BrCA
**Leucine**	Leu	**157.89 ± 39.4**	**105.38 ± 40.2**	1.50	*0.013*	Higher in BrCA
**Ornithine**	Orn	**115.55 ± 35.5**	**93.53 ± 36.1**	1.24		
**Lysine**	Lys	**342.37 ± 119.9**	**250.04 ± 89.5**	1.37		

Serum concentrations are shown as means and standard deviations. All p-values are adjusted for both age and BMI by linear regression. A P-value <0.05 was considered significant.

**Table 3:** Amino acid concentrations in postoperative serum

**Amino acid**		**Serum concentration [μg/mL]**		**Fold difference**	***P-value***	**Trend**
		**BrCA [n = 38]**	**HD [n = 10]**			
**Aspartic acid**	Asp	**22.51 ± 8.4**	**21.16 ± 7.9**	1.06		
**Glutamic acid**	Glu	**188.19 ± 101.8**	**96.71 ± 35.6**	1.95	*0.037*	Higher in BrCA
**Asparagine**	Asn	**69.50 ± 25.3**	**67.43 ± 15.6**	1.03		
**Serine**	Ser	**220.99 ±73.4**	**225.64 ± 43.7**	0.98		
**Glutamine**	Gln	**632.40 ± 146.7**	**703.62 ± 126.3**	0.90		
**Citrulline**	Cit	**55.67 ± 19.1**	**43.22 ± 8.4**	1.29		
**Arginine**	Arg	**210.54 ± 57.8**	**180.39 ± 38.5**	1.17		
**Alanine**	Ala	**795.24 ± 257.6**	**648.57 ± 205.4**	1.23		
**Tyrosine**	Tyr	**123.16 ± 44.5**	**96.26 ± 25.0**	1.28		
**Valine**	Val	**411.64 ± 123.4**	**320.08 ± 35.8**	1.29	*0.037*	Higher in BrCA
**Phenylalanine**	Phe	**117.79 ± 31.5**	**106.87 ± 22.4**	1.10		
**Isoleucine**	Ile	**118.02 ± 48.3**	**85.11 ± 17.5**	1.39		
**Leucine**	Leu	**218.70 ± 81.0**	**170.95 ± 35.1**	1.28		
**Ornithine**	Orn	**157.55 ± 44.6**	**160.92 ± 39.8**	0.98		
**Lysine**	Lys	**451.55 ± 146.7**	**478.04 ± 101.1**	0.94		

Serum concentrations are shown as means and standard deviations. All p-values are adjusted for both age and BMI by linear regression. A P-value <0.05 was considered significant.

### Amino acid levels increase after surgery

Table 4 and Additional file 2: Figure S1 show the fold changes from pre- to postoperative amino acid serum levels for HD and BrCA separately. Twelve of 15 amino acids levels increased significantly in both groups when comparing pre- and postoperative values, with fold increases ranging between 1.30 and 2.03. Significantly higher fold changes in HD, after adjustment for age and BMI, were seen in 3 out of 15 amino acids (Asp, Gln and Phe).

**Table 4:** Changes in pre- to postoperative amino acid levels in healthy donors (HD) and breast cancer patients (BrCA)

**Amino acid**		**Fold difference pre- to postoperative**				***P-value (group difference)***
		**BrCA [n = 38]**	***p***	**HD [n = 9]**	***p***	
**Aspartic acid**	Asp	**1.08 ± 0.51**	*ns*	**1.68 ± 0.81**	*0.013*	***0.049***
**Glutamic acid**	Glu	**1.19 ± 0.57**	*ns*	**1.20 ± 0.33**	*ns*	
**Asparagine**	Asn	**1.30 ± 0.43**	*0.000*	**1.48 ± 0.37**	*0.001*	
**Serine**	Ser	**1.35 ± 0.47**	*0.000*	**1.77 ± 0.65**	*0.000*	
**Glutamine**	Gln	**1.19 ± 0.47**	*ns*	**1.89 ± 0.44**	*0.000*	***0.004***
**Citrulline**	Cit	**1.41 ± 0.53**	*0.000*	**1.38 ± 0.47**	*0.030*	
**Arginine**	Arg	**1.50 ± 1.23**	*0.000*	**1.60 ± 0.52**	*0.002*	
**Alanine**	Ala	**1.44 ± 0.50**	*0.000*	**1.90 ± 0.58**	*0.000*	
**Tyrosine**	Tyr	**1.39 ± 0.57**	*0.000*	**1.75 ± 0.77**	*0.028*	
**Valine**	Val	**1.35 ± 0.49**	*0.001*	**1.73 ± 0.59**	*0.000*	
**Phenylalanine**	Phe	**1.32 ± 0.39**	*0.000*	**1.87 ± 0.64**	*0.000*	***0.030***
**Isoleucine**	Ile	**1.30 ± 0.52**	*0.007*	**1.68 ± 0.72**	*0.003*	
**Leucine**	Leu	**1.42 ± 0.49**	*0.000*	**1.81 ± 0.69**	*0.000*	
**Ornithine**	Orn	**1.51 ± 0.78**	*0.000*	**1.95 ± 0.95**	*0.001*	
**Lysine**	Lys	**1.61 ± 1.61**	*0.000*	**2.03 ± 0.60**	*0.000*	

Fold differences are represented by means and SD. P-values in the respective groups represent the difference between pre- and postoperative value per amino acid; p-values in bold style (right column) represent significant differences in fold change, adjusted for age and BMI, between the two groups (HD and BrCA). ns: not significant. A P-value <0.05 was considered significant.

Pre- to postoperative fold changes were not dependent on the extent of surgery performed as values in patients operated with breast-conserving surgery did not differ from values in those having a mastectomy.

### Preoperative amino acid levels differ significantly between molecular tumor subtypes

Gene expression arrays have led to the identification of fundamentally different molecular subtypes of breast cancer [19]. For practical purposes, the 12th St Gallen International Expert Consensus on breast cancer [17] recently set up histopathological surrogate parameters based on immunohistochemical analysis of hormone receptor status, Her2-neu amplification and proliferation index according to Ki67 labeling. The subtypes identified thus far (luminal A, luminal B (Her2 negative), luminal B (Her2 positive), Her2 over-expressing (non-luminal) and basal-like (triple negative)) differ significantly in prognosis and prediction of treatment response.

**Figure 2:**
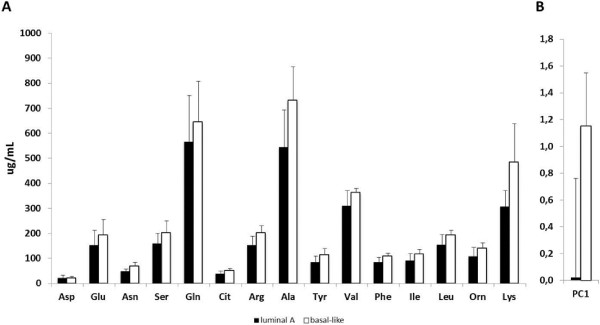
**Amino acid levels in patients with luminal A or basal-like breast cancer. (A)** Blood amino acid levels and **(B)** principal component (PC) 1 values in the prognostically most (luminal A, n = 19) and least (basal-like, n = 6) favorable molecular subtypes of breast cancer.

In the present study, Her2-positive luminal B (n = 1) and Her2 over-expressing non-luminal (n = 2) tumors were under-represented and thus not included in the analysis. Comparing the remaining three subtypes, significant differences were found in 5 of 15 amino acids (Asn, Ala, Iso, Leu and Lys; p = 0.008, 0.041, 0.008, 0.009 and 0.043, respectively) and PC1 (p = 0.015). After further analysis, the difference clearly resided in a marked difference in amino acid levels between the prognostically most (luminal A, n = 18) and least (basal-like, n = 6) favorable group. Patients with basal-like tumors had significantly higher preoperative amino acid levels than patients with luminal A tumors in all but three amino acids (Asp, Gln and Glu), confirmed by PC1 (p = 0.002), see Figure 2A and B.

There were no differences in preoperative amino acid serum levels between different tumor stages or node-positive *versus* node-negative patients.

### Preoperative amino acids levels correlate with pro-inflammatory parameters

PC1 correlated with pro-inflammatory parameters measured in the same material, namely a Th1-like profile defined as CCR7-CCR5 + CXCR3+ (R .418, p = 0.027) and the production of IFN-γ and TNF-α in CD4+ T lymphocytes (R .419 and .402 with p = 0.003 and 0.005, respectively). At the same time, negative correlations with potentially suppressive subgroups of myeloid cells (Lin-DR-CD33+ and Lin-DR-CD34+) were observed (R -.369 and -.384 with p = 0.008 and 0.006, respectively). Interestingly, the expression of Fas ligand on CD8+ T lymphocytes also showed a significant negative correlation with PC1 (R -.626, p = 0.002).

### Performance of a principal component as predictive tool of health state

**Figure 3:**
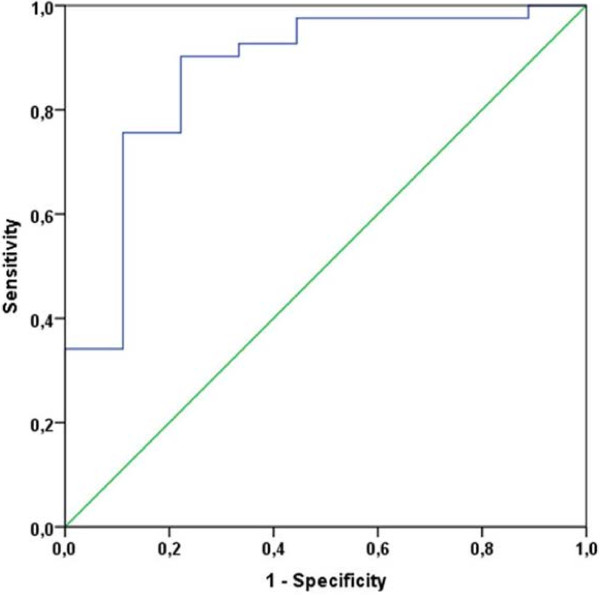
Receiver operating characteristic curve for principal component (PC) 2 as diagnostic tool for health state at a cut-off value of −0.75.

For the hypothesis-generating evaluation of a potential predictive value of amino acid levels for health state, a second principal component (PC2) was generated, containing those eight amino acids that were significantly different between HD and BrCA preoperatively. The mean value of PC2 was −1.12 (±0.90) for HD and 0.25 (±0.85) for BrCA (p = 0.000). A receiver operating characteristic (ROC) curve was drawn (see Figure 3). The area under the curve (AUC) was 0.870 (95% CI 0.728-1.000; p = 0.001), suggesting good discriminatory power. After optimal binning of PC2 for the distinction of health state, a cut-off value −0.75 was established. This correctly identified 7/9 healthy women (specificity 78%) and 37/41 breast cancer patients (sensitivity 90%), resulting in a false negative rate of 9.7%.

## Discussion

There are few, and mostly small, studies reporting on plasma or serum levels of amino acids, with contradictory results. In 22 breast cancer patients, Kubota et al. reported an increase of total amino acid levels, with decreased levels of cysteine and glutamine, but increased levels of alanine, arginine and threonine [20]. Another study [21] evaluated 33 breast cancer patients and observed significant increases of ornithine, glutamic acid and free tryptophan. Proenza et al. [22] demonstrated a decreased level of aspartic acid, but increased levels of asparagine, glutamine and hydroxyproline in 16 breast cancer patients. Miyagi et al. could show an increase in the plasma free amino acids threonine, serine, proline, glycine, alanine, ornithine and lysine, and a decrease of glutamine, tyrosine, histidine and tryptophane in 196 breast cancer patients in comparison with 976 controls [4]. In a more recent publication from the same group, in which the discriminative diagnostic value of AA levels was tested, the reported predictive potential was high enough to warrant clinical use [5]. Similarly to the group’s earlier report, levels of individual AA were noted to be either increased or decreased in comparison with healthy controls, and three potential predictive indexes were created using selected amino acids. In an extensive metabolomics analysis on tissue samples from 271 breast cancer patients, Budczies et al. [23] could recently show an altered metabolic phenotype compared with normal breast tissue. Changes in amino acid metabolism involved an at least 1.9-fold increase in 16 proteinogenic amino acids in cancerous compared with normal tissue, unchanged levels of arginine and glutamine, and a decrease in asparagine. Thus, there seems to be a clear effect of breast cancer on metabolic variables, however, the pattern of changes varies, probably owing to different measurement techniques applied, lack of data adjustment for potential confounders, and biological material examined.

In the current study, reported differences between healthy donors and breast cancer patients were rigorously adjusted for the two potentially strong confounding factors, BMI and age. This established robust outcomes pointing towards an independent cancer-related effect. This assumption was supported by the fact that amino acid levels were significantly higher in the most (basal-like) than the least (luminal A) aggressive intrinsic tumor subtype. In addition, 13 of 15 AA levels were indistinguishable between HD and BrCA after surgical tumor removal.

Interestingly, most amino acid levels increased after surgery. This effect was not related to the extent of surgery as defined by breast-conserving surgery or lumpectomy *versus* mastectomy. One possible explanation could be the presence of physiological postoperative inflammation as a part of the wound healing process. It is unclear, however, why the increase in AA from pre- to postoperative levels was higher in HD than BrCA. Minet-Quinard et al., who described a normalization of three preoperatively increased amino acids after surgical tumor removal, did not observe any rise in amino acid levels, however, only three amino acids were studied in this respect [24].

Even though several publications describe changes in amino acid levels in cancer patients versus healthy controls, explanatory models are scarce. In some cancers, decreased levels of selected amino acids have been interpreted as the results of high-demand tumor metabolism associated with early signs of malnutrition. The fact that none of the 15 measured amino acids in this study showed decreased levels in comparison with healthy donors suggests that the tumor stage in the study population was not advanced enough for the tumor burden to diminish the amino acid pool. This notion of a population with mostly early-stage breast cancer is supported by the fact that the vast majority (70.7%) was node negative and the median tumor size was only 16 mm (Table 1). Increased levels of serine may be explained by the increased enzymatic activity involved in serine biosynthesis in tumor cells [25]. The increased glutamate levels could be interpreted as a sign of increased glutamine metabolism in the tumor, however, this does not explain the parallel rise of glutamine [26]. Alanine, glutamate, serine, glycine and aspartate may be produced by tumor cells themselves [27]. It would clearly be of significant interest to perform a similar analysis on the sera of patients with late-stage breast cancer in order to compare AA levels to the results from the current cohort under the assumption that more advanced disease might instead lead to the consumption of AA.

In addition to the nutritional demands of the tumor, an important starting point of this study was the expected depletion of L-arginine through immune cells, based on the increase in myeloid cells over-expressing the enzyme arginase 1 in the same patient cohort [14]. However, this was not the case. The principal component (PC1) representing preoperative AA levels correlated instead with a pro-inflammatory immunological profile. The somewhat surprising association of the PC1 with Fas-ligand expression on cytotoxic T-cells may support the earlier notion of a mixed signature of activation and suppression in T-cells from early-stage breast cancer patients [16].

As shown in several publications [4,28], AA levels have the potential to be used as diagnostic tools discriminating between cancer patients and healthy subjects. The small sample size in the present study served as a pilot investigation showing a second principal component (PC2) with good accuracy as a diagnostic test which could potentially be developed further. This may serve as a hypothesis-generating finding which needs to be validated in a larger patient cohort.

## Conclusions

This prospective study of 41 breast cancer patients (BrCA) and nine healthy donors (HD) with benign breast lesions showed significantly higher serum levels in eight out of 15 amino acids in BrCA than HD. Increased amino acid levels correlated with several pro-inflammatory immunological factors and a more aggressive intrinsic tumor subtype. Creating a principal component of significantly different amino acids and testing it for prediction of health state, a threshold value was defined resulting in a good discriminatory power between healthy donors and breast cancer patients with potential utility as a diagnostic test. These findings warrant validation in a larger cohort.

## Competing interests

The authors declare that they have no competing interests.

## Authors’ contributions

This study was designed by JB, IP and RK. Patient enrolment was carried out by JB, data acquisition and flow cytometry analysis by IP and YM. Statistical analysis and interpretation was performed by JB and IP, who also drafted the first version of this manuscript. All authors critically revised the text and gave their approval of the final manuscript.

## Supplementary Material

Additional file 1: Table S1 Antibodies used in flow cytometry. Click here for file

Additional file 2: Figure S1 Raw data analysis of all 15 serum amino acids (A-O) in individual healthy controls **(a)** and breast cancer patients **(b)** before and after surgery. Click here for file
