# Supplementary material for: Tumor-dependent increase of serum amino acid levels in breast cancer patients has diagnostic potential and correlates with molecular tumor subtypes
Source: J Transl Med. 2013 Nov 16;11:290. doi: 10.1186/1479-5876-11-290 (PMC3835137; doi:10.1186/1479-5876-11-290)
Supplement: Additional file 1: Table S1 — Antibodies used in flow cytometry. [file 1479-5876-11-290-S1.docx]

**Supplemental Table 1:** Antibodies used in flow cytometry

| **Marker** | **Conjugate** | **Clone** |
| --- | --- | --- |
| **CD3** | PE-Cy7 ^c^ | UCHT1 |
|  | PerCP-Cy5.5^a^ | SK7 |
| **CD4** | PE-Alf610 ^d^ | S3.5 |
|  | PerCP-Cy5.5^a^ | SK3 |
|  | Fitc ^c^ | Sk3 |
| **CD8** | PE-Alf610 ^d^ | 3B.5 |
|  | APC-Cy7 ^c^ | RPA-T8 |
| **CD14** | Pacific Blue^a^ | M5E2 |
| **CD19** | PerCP-Cy5.5^a^ | SJ25C1 |
| **CD33** | APC^f^ | CD33-403 |
| **CD34** | PE-Cy7^a^ | 8G12 |
| **CD95 (FasL)** | APC^d^ | Alf-2.1 |
| **CD183 (CXCR3)** | Pacific Blue ^c^ | TG1/CXCR3 |
| **CD195 (CCR5)** | Fitc ^c^ | HEK/1/85a |
| **CD197 (CCR7)** | PE ^a^ | 150503 |
| **IFNγ** | PE ^a^ | 4S.B3 |
| **HLA-DR** | PE^a^ | L243 |
| **TNFα** | APC ^a^ | 6401.1111 |

*^a^ BD Biosciences (San Jose, CA, USA), ^b^ Beckman Coulter (High Wycomb, UK), ^c^ Biolegend (San Diego, CA, USA), ^d^ Invitrogen (Carlsbad, CA, USA), ^e^ eBioscience (San Diego, CA, USA), ^f^ Caltag (Buckingham, UK)*
