# Supplementary figures and images for: Tumor-dependent increase of serum amino acid levels in breast cancer patients has diagnostic potential and correlates with molecular tumor subtypes
Source: J Transl Med. 2013 Nov 16;11:290. doi: 10.1186/1479-5876-11-290 (PMC3835137; doi:10.1186/1479-5876-11-290)

Supplemental Figure 1

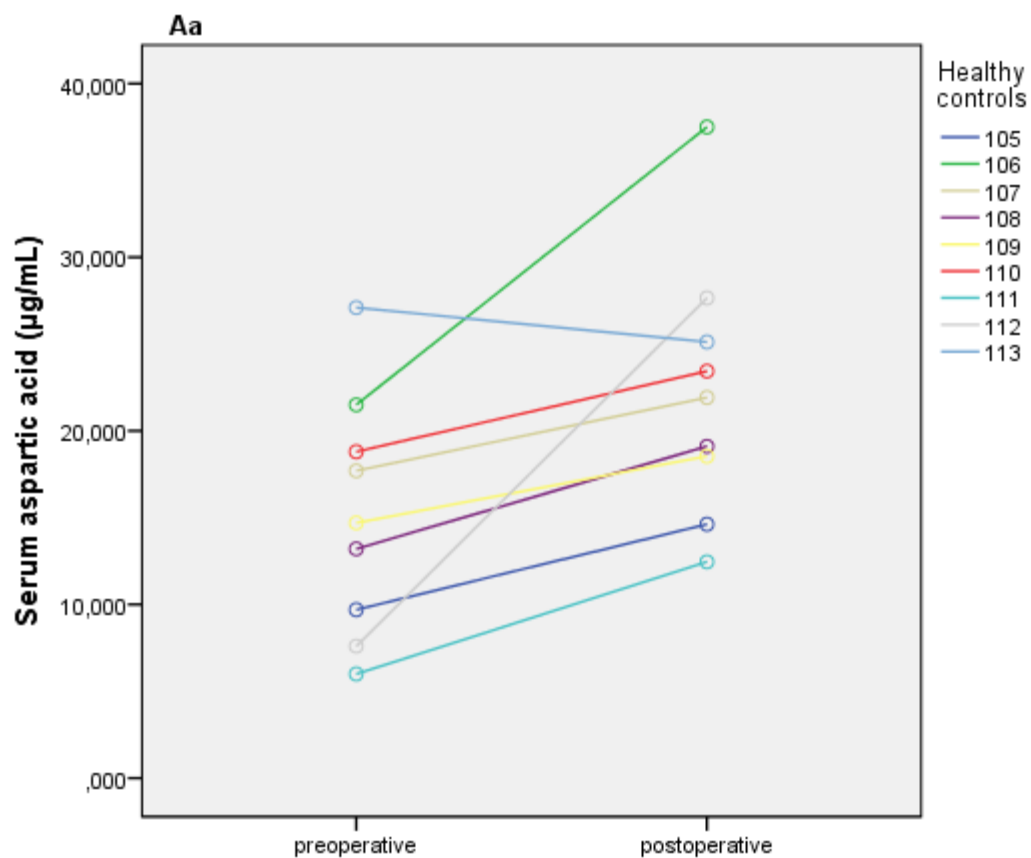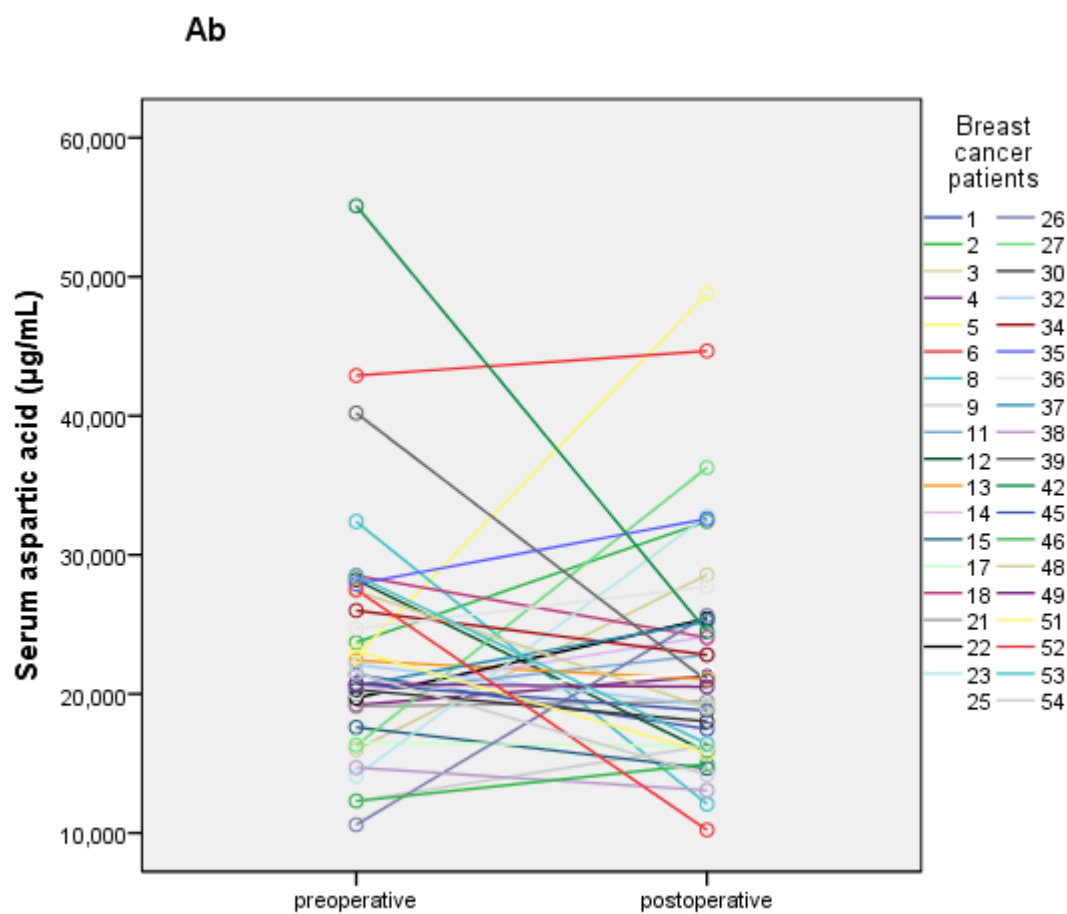

**Ba**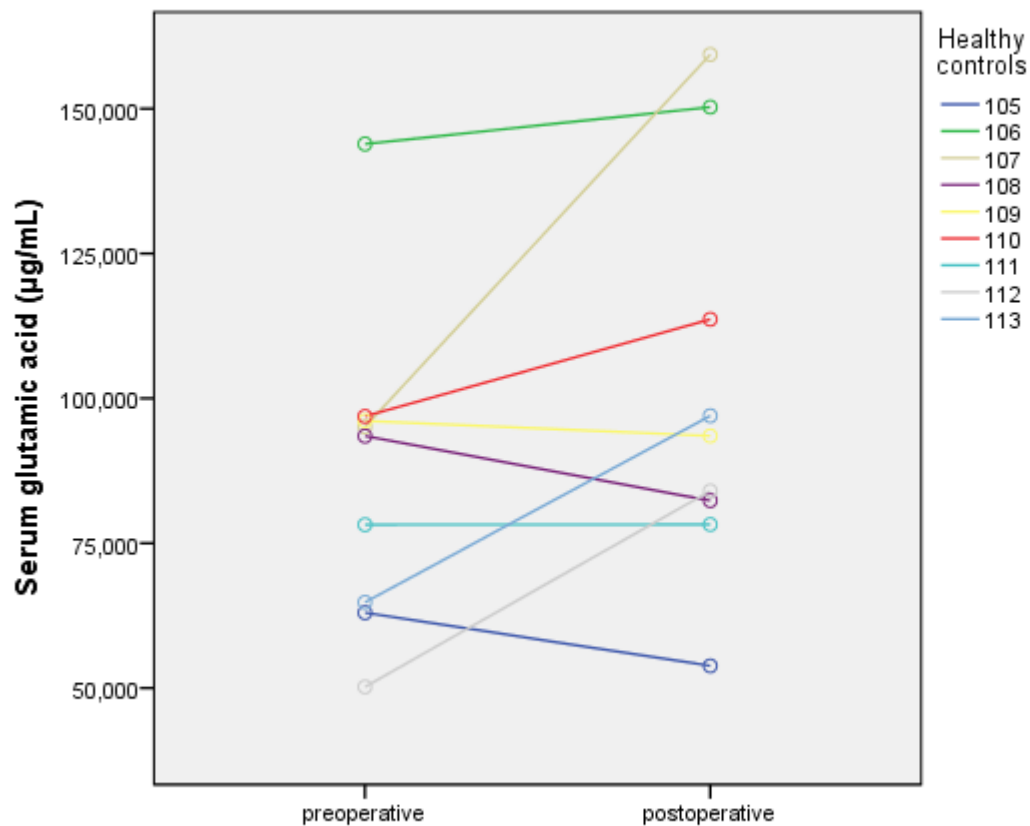**Bb**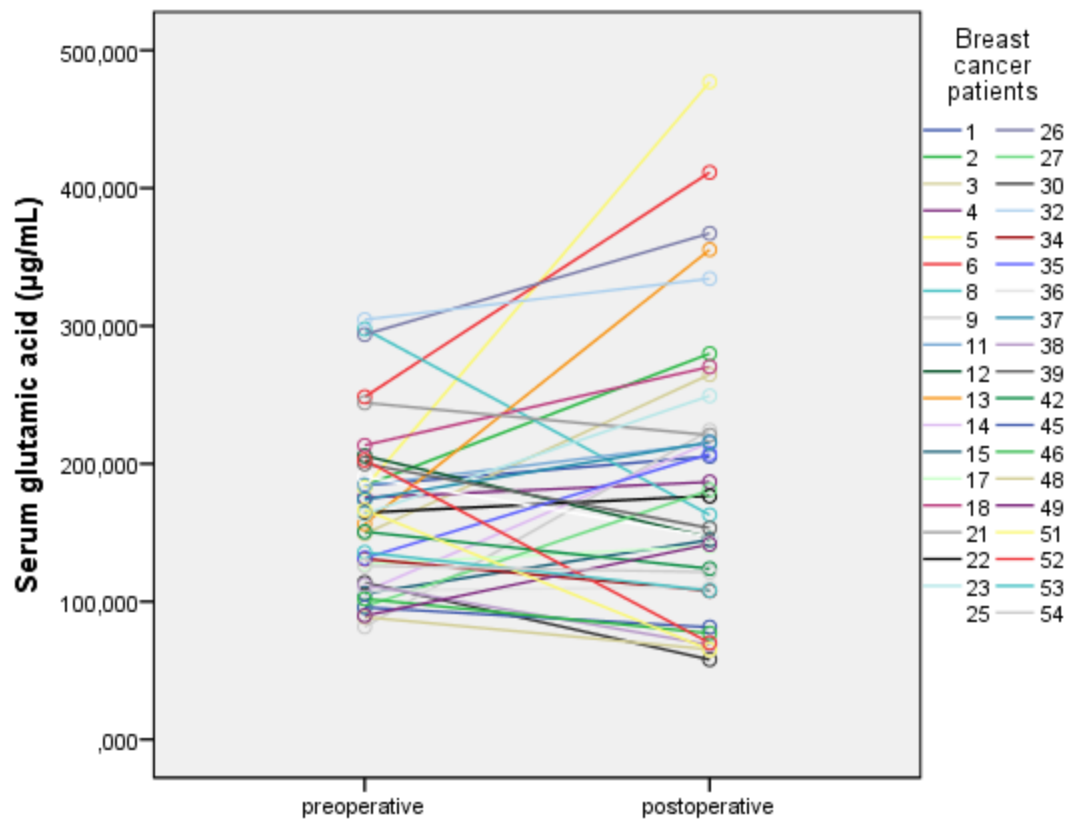

**Ca**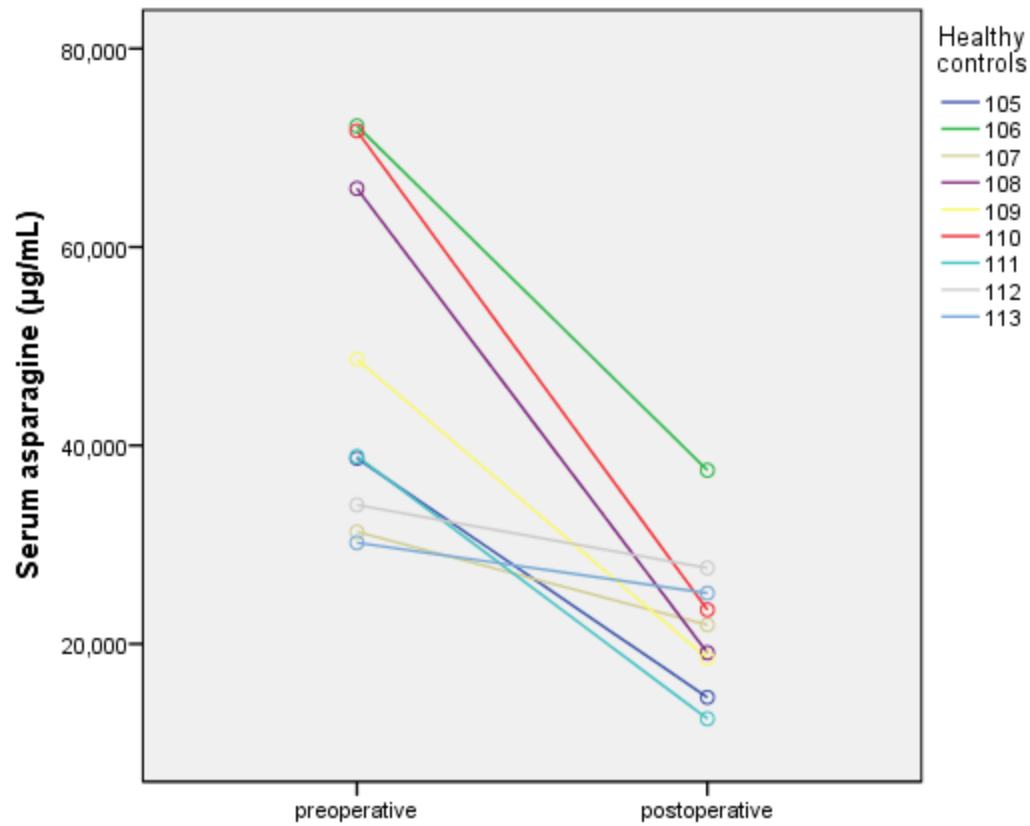**Cb**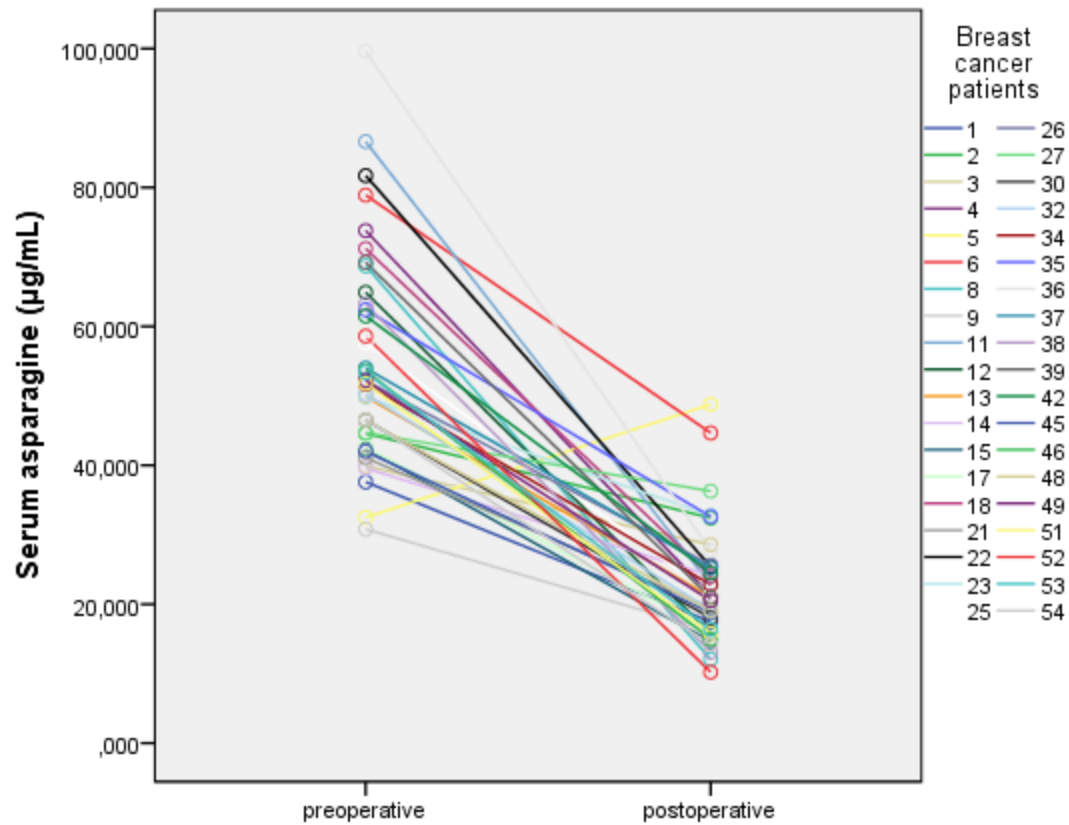

**Da**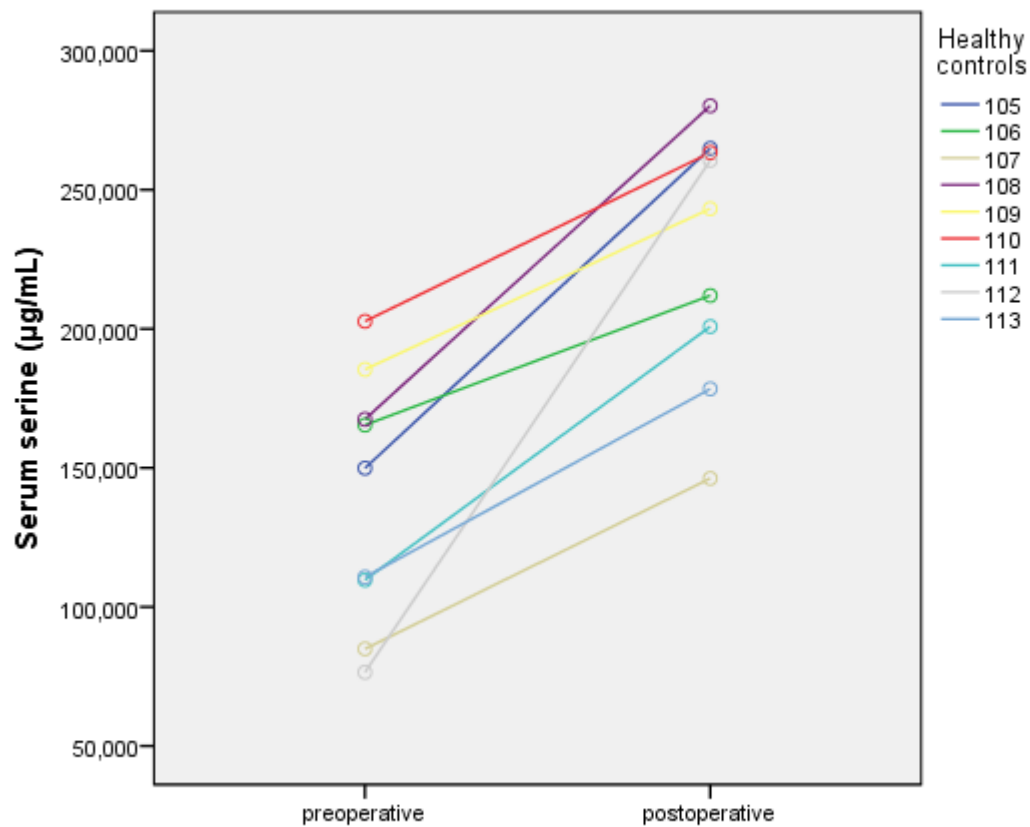**Db**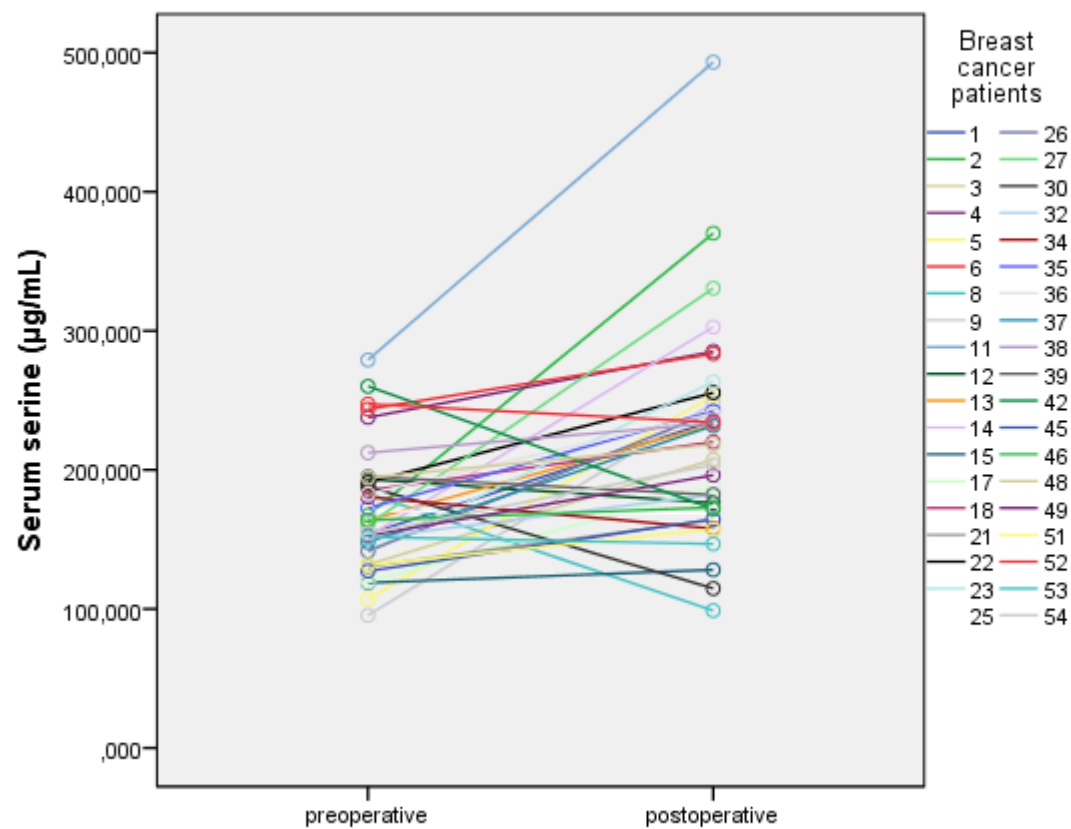

**Ea**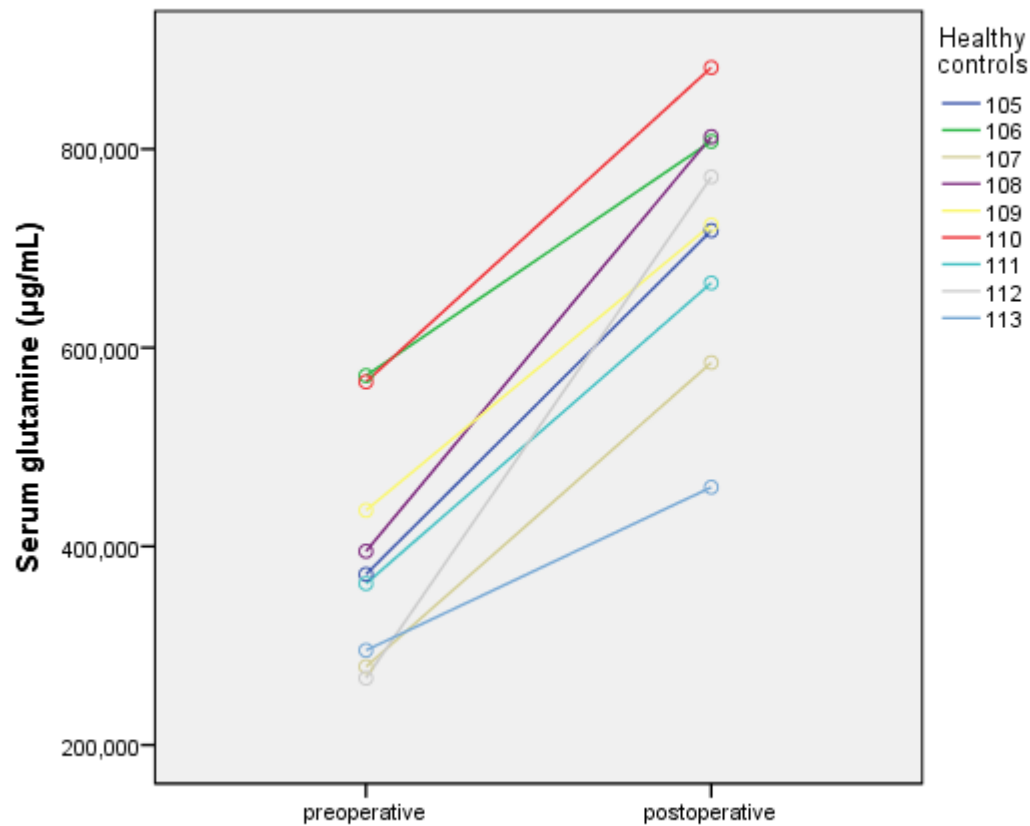**Eb**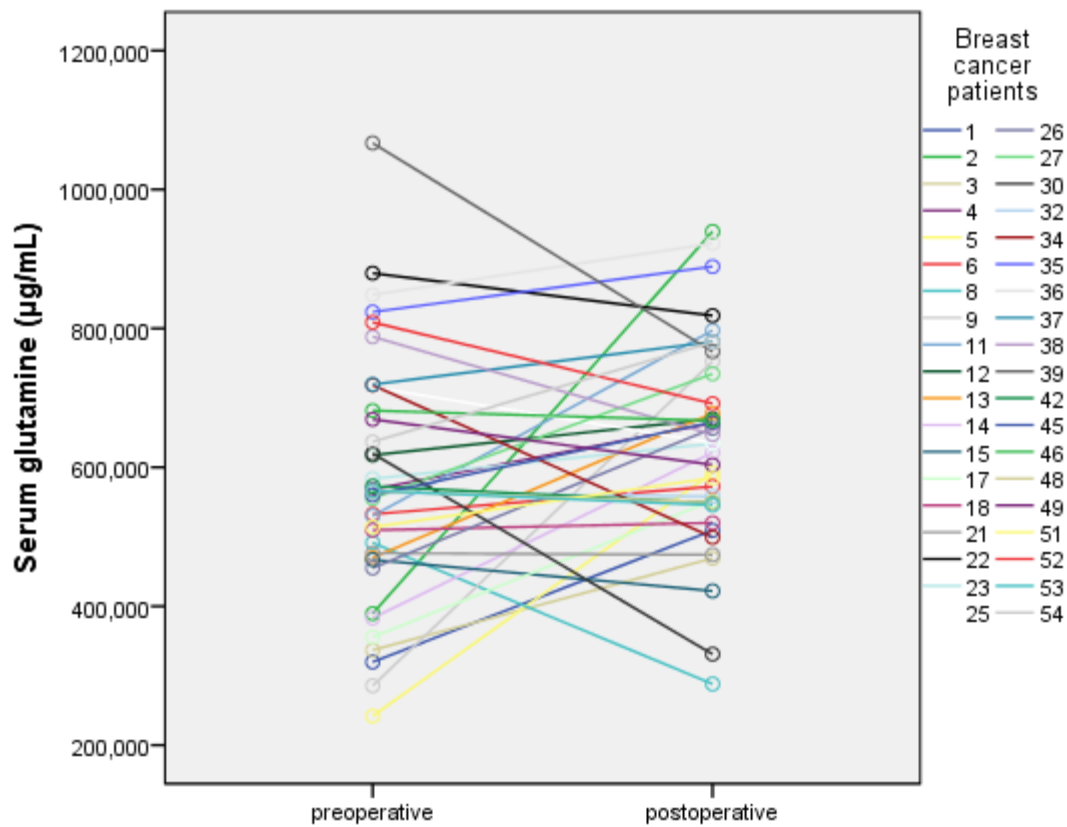

**Fa**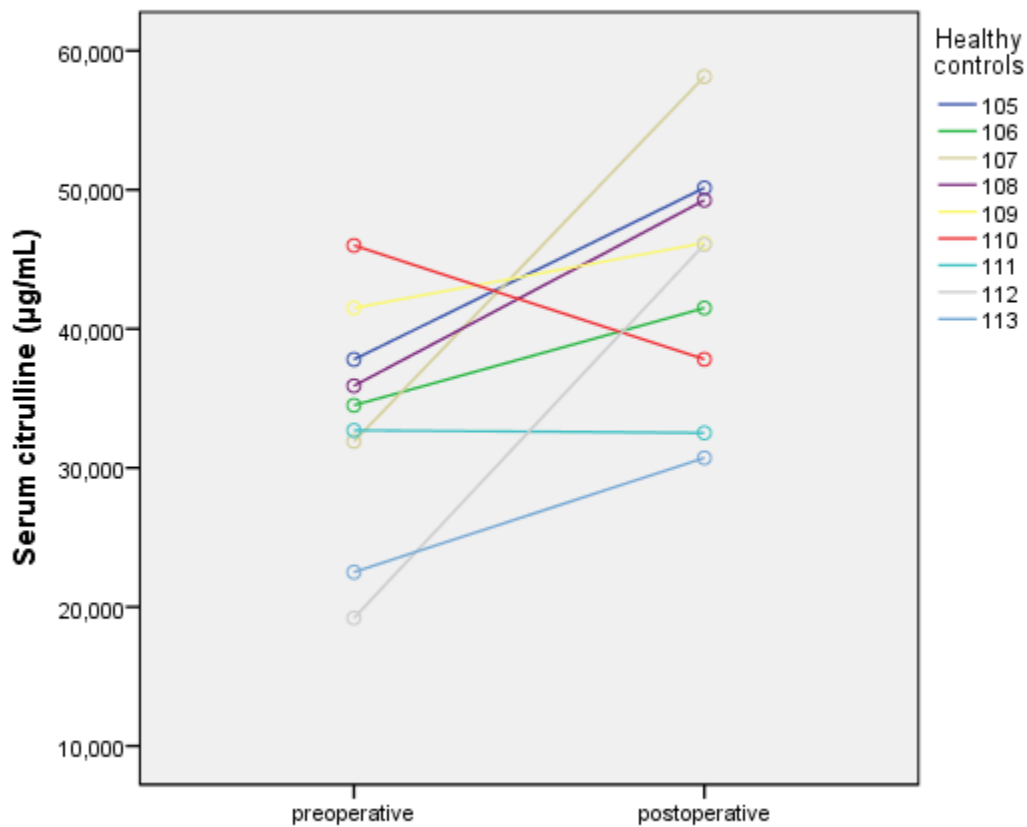**Fb**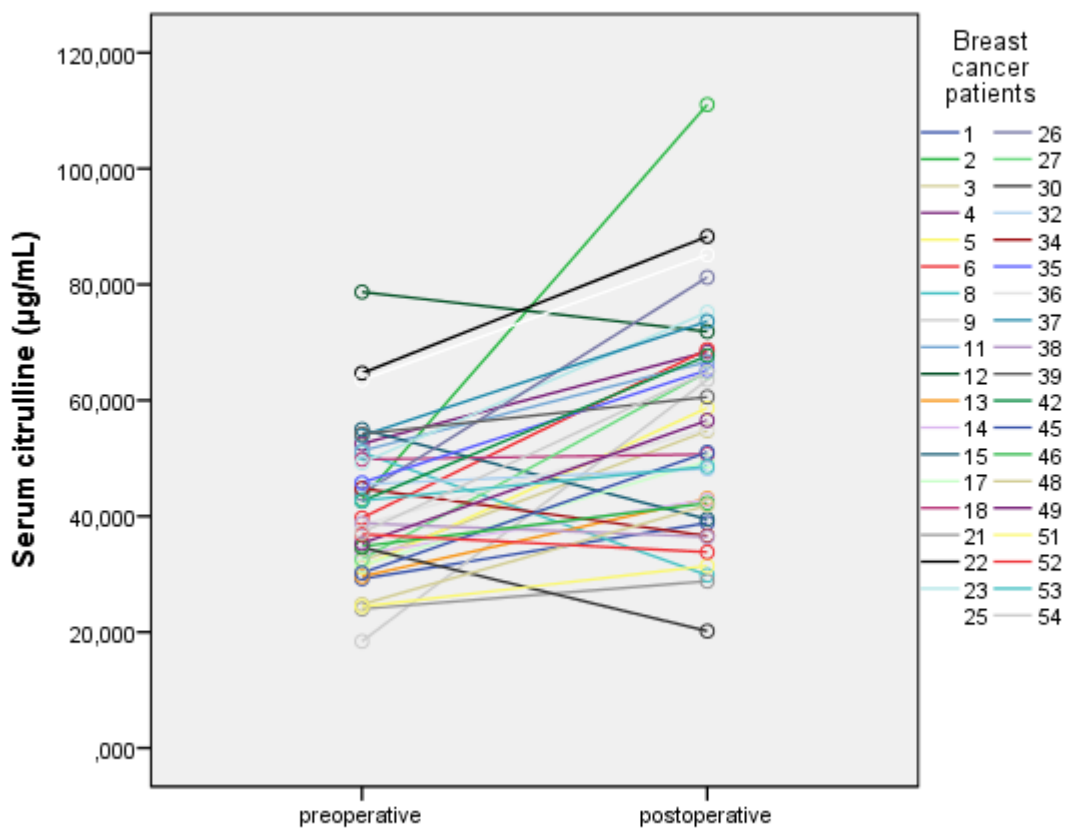

**Ga**

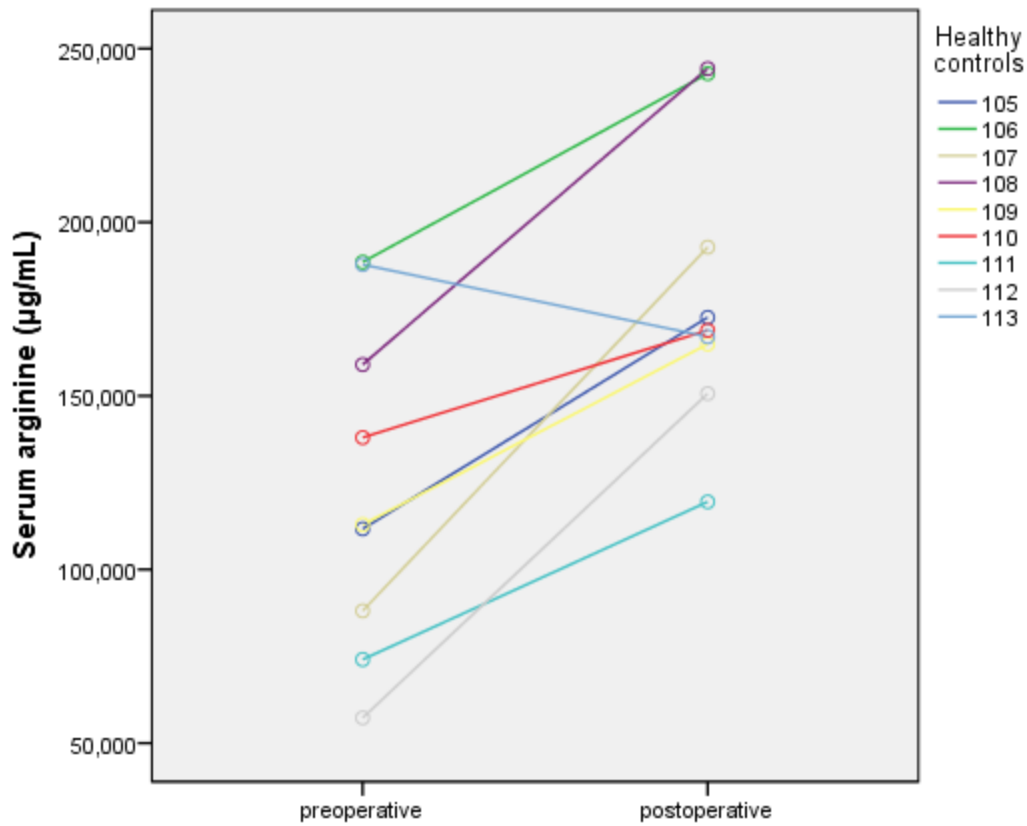

**Gb**

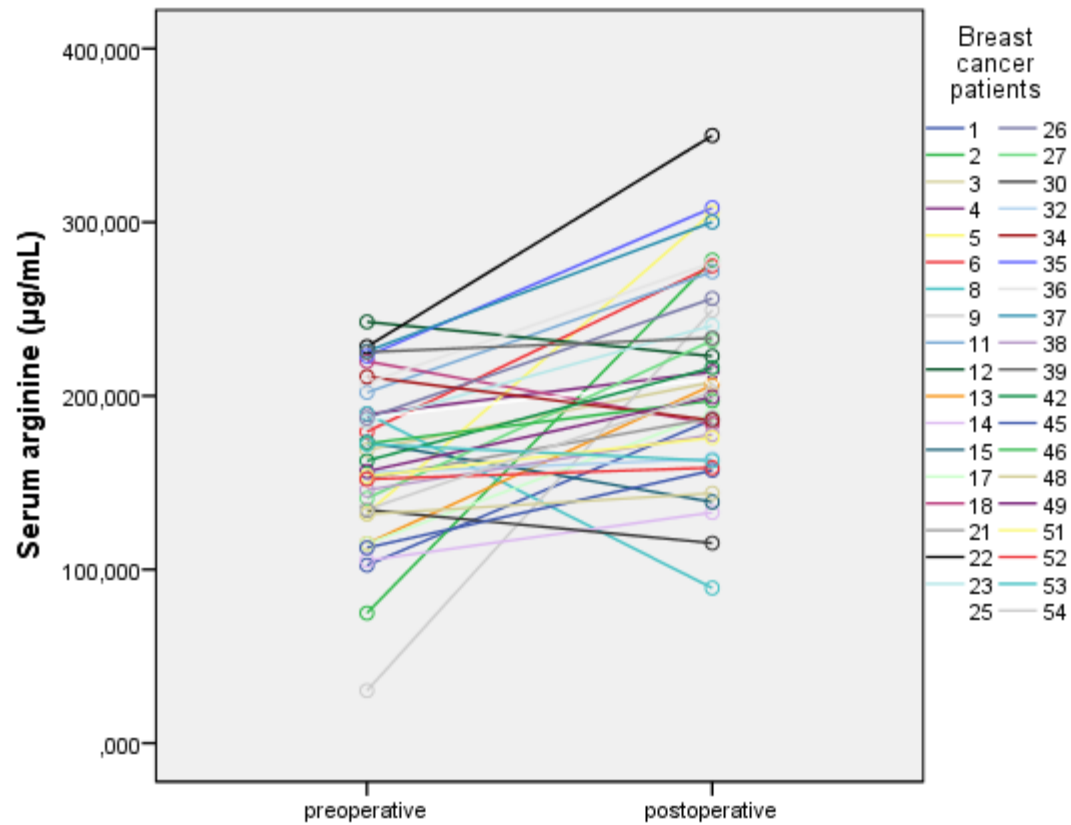

**Ha**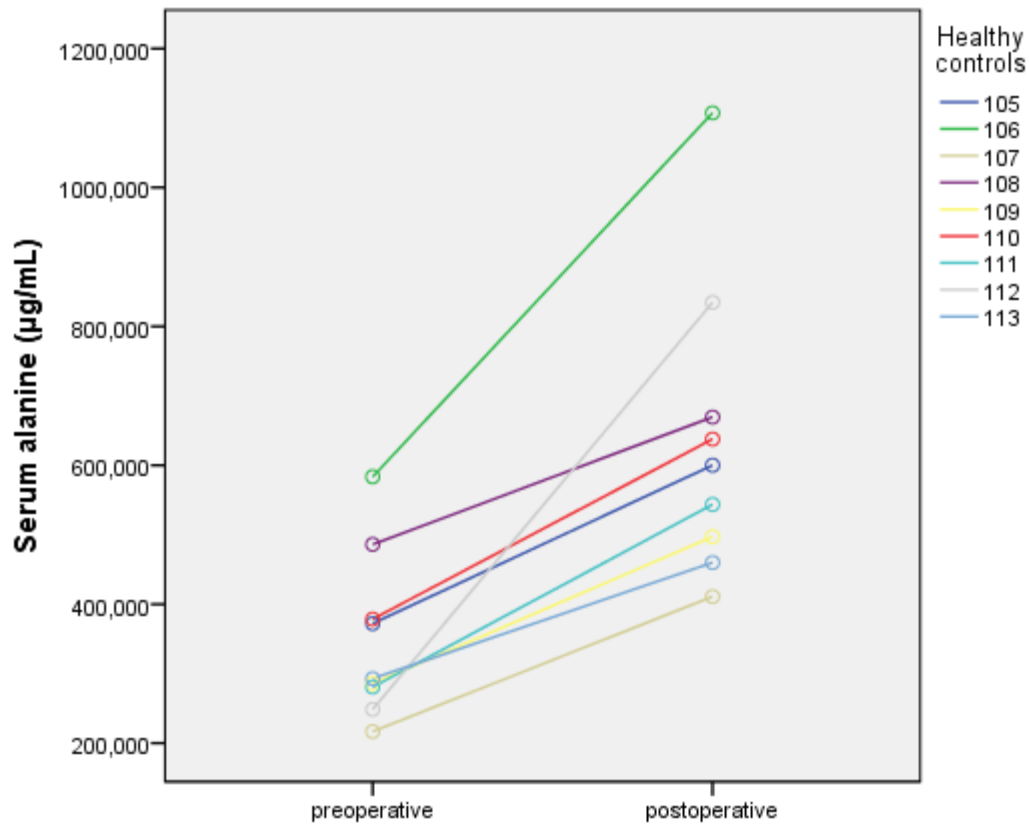**Hb**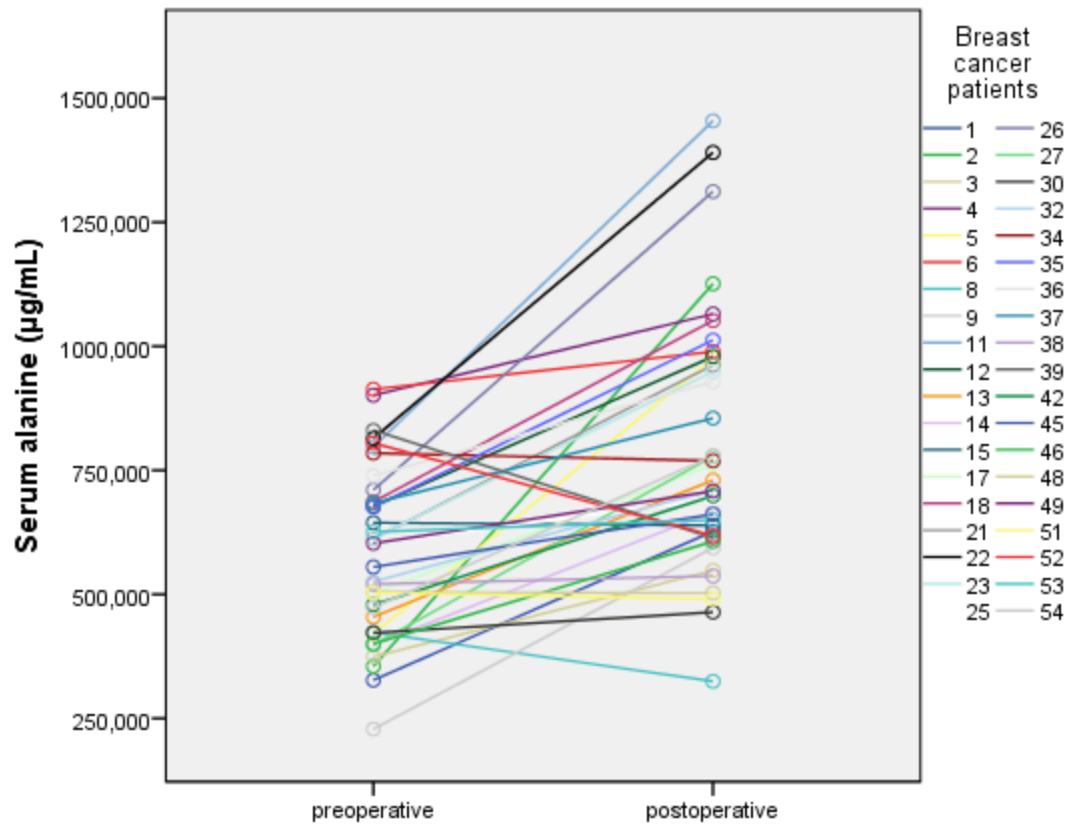

**1a**

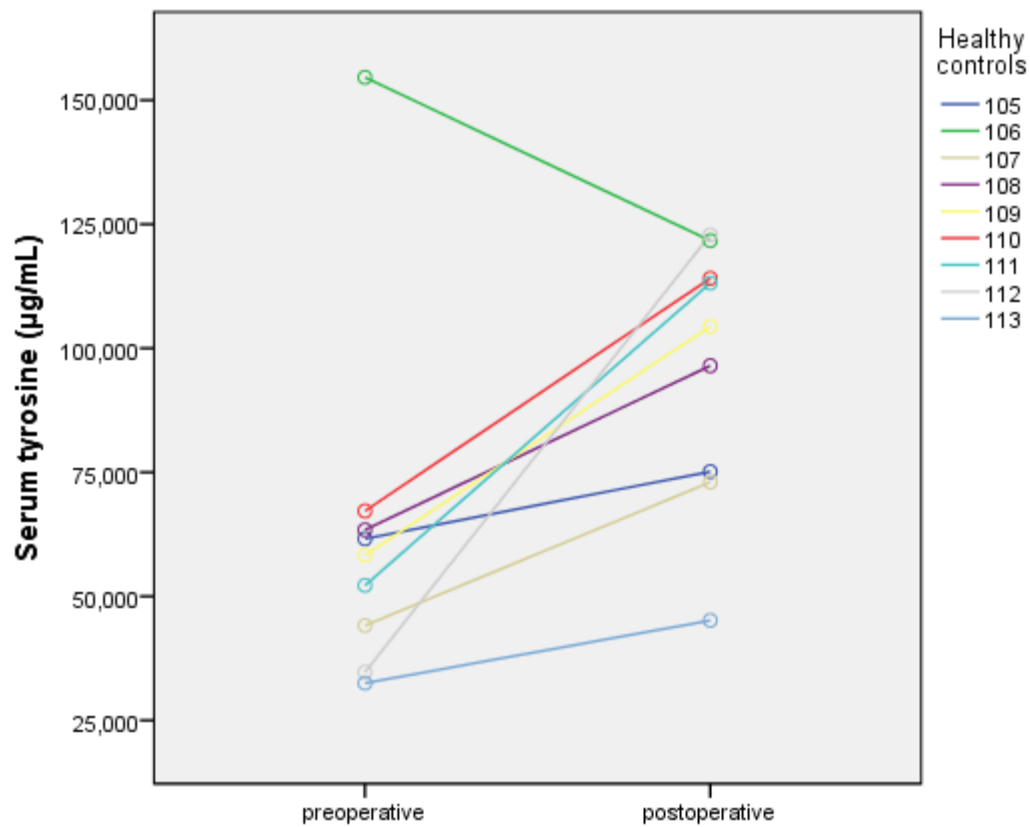

**1b**

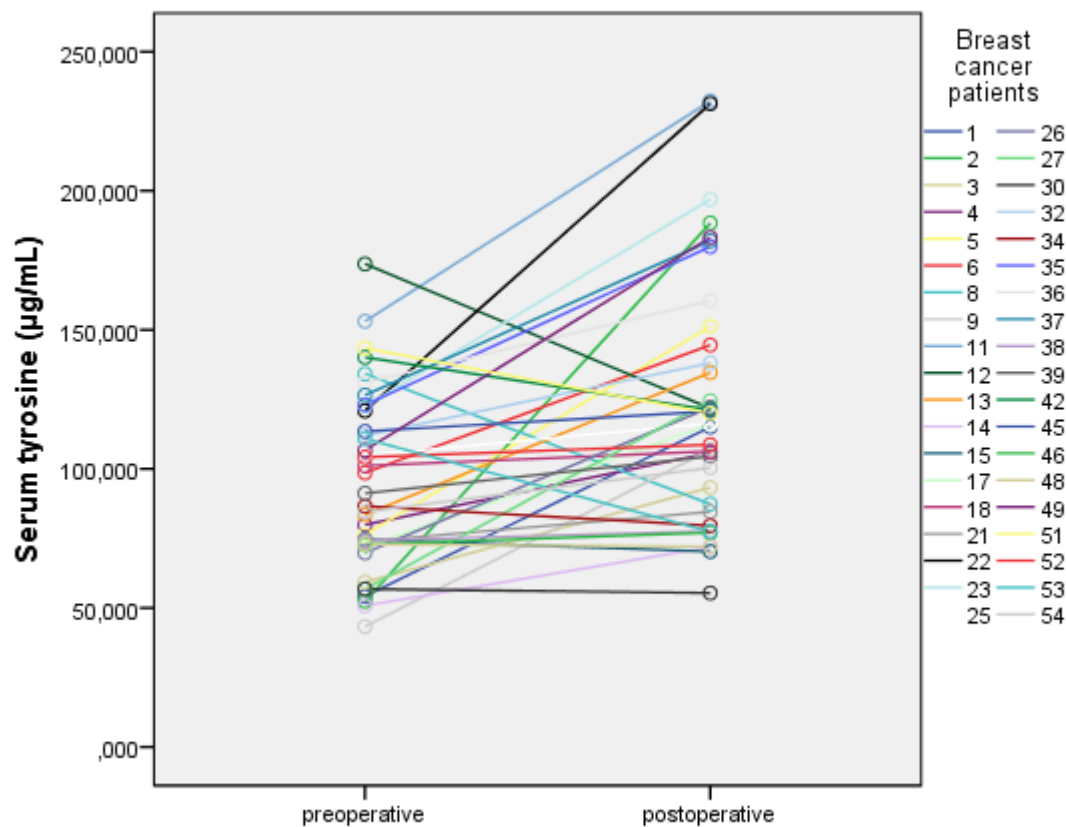

**Ja**

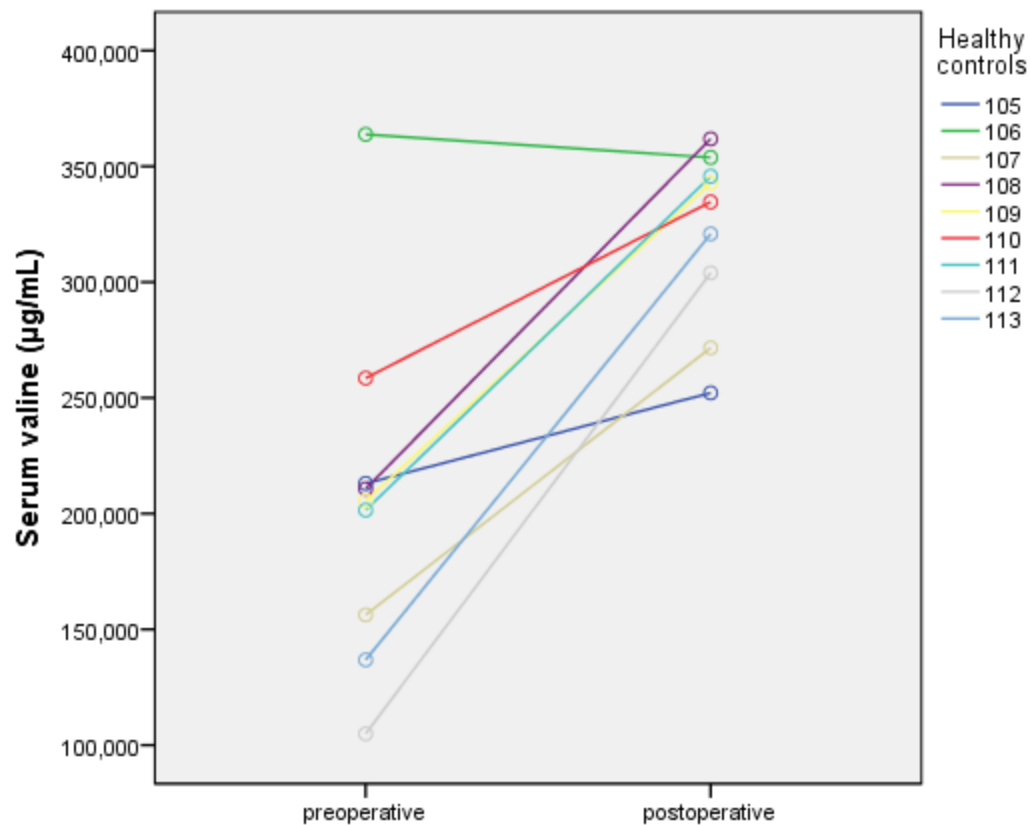

**Jb**

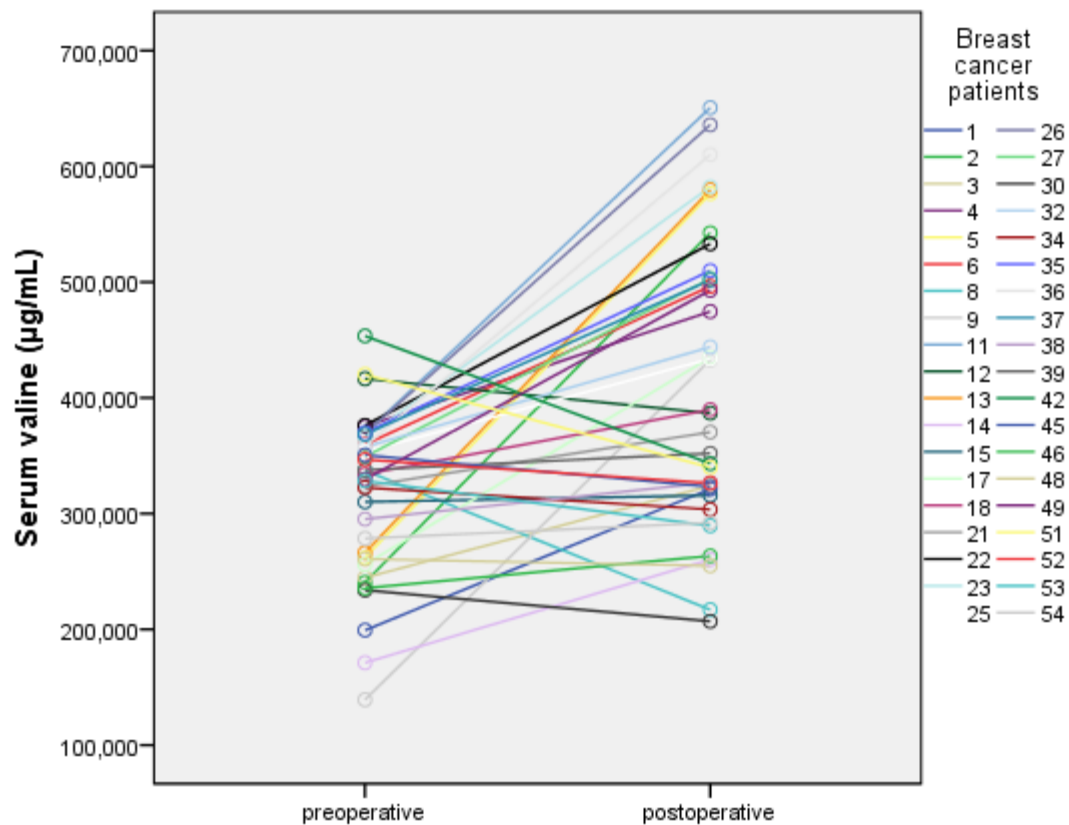

**Ka**

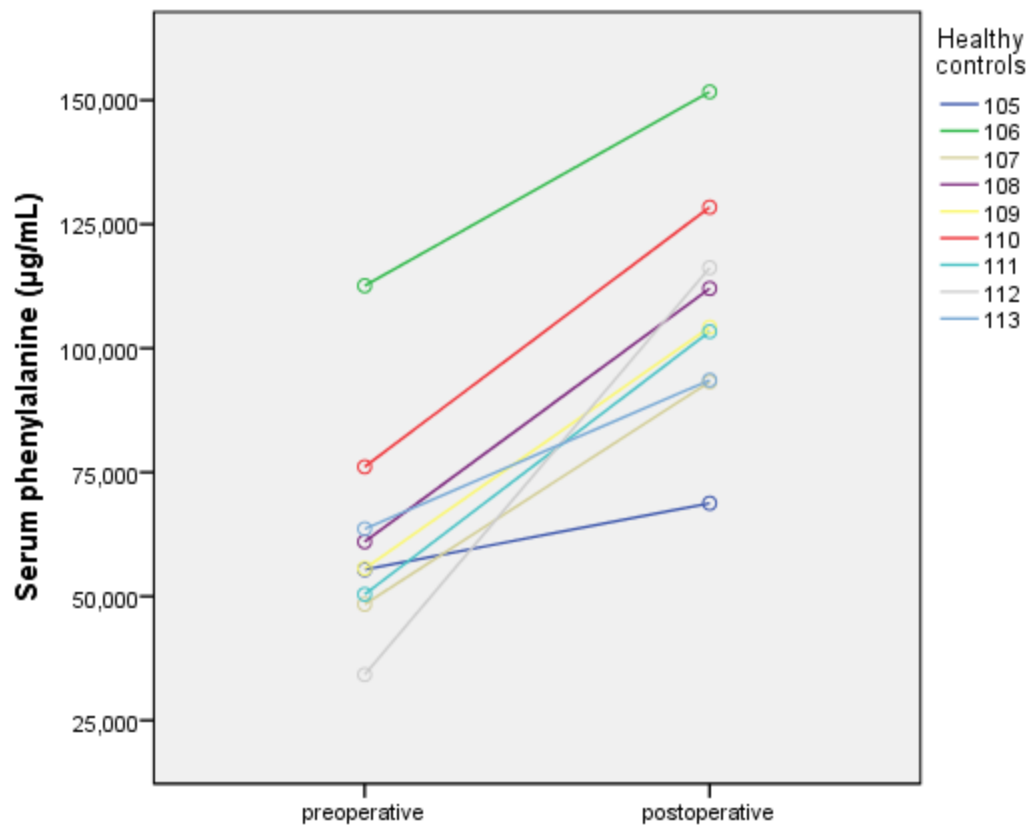

**Kb**

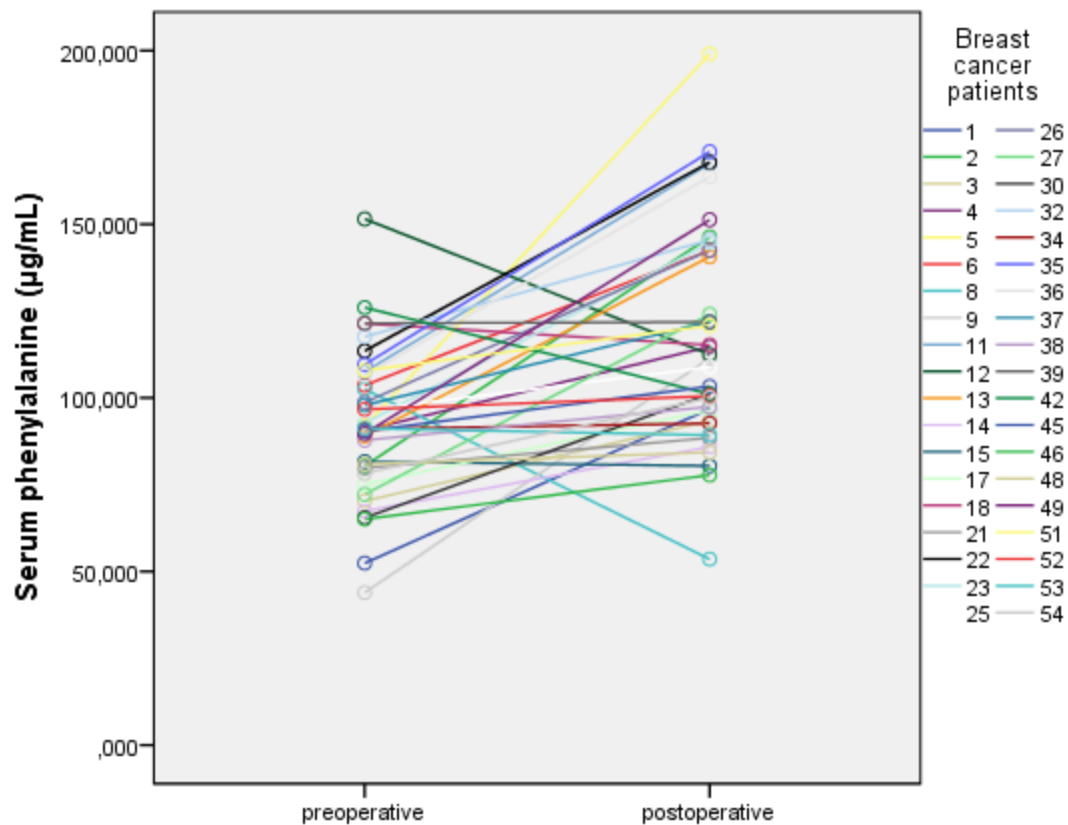

**La**

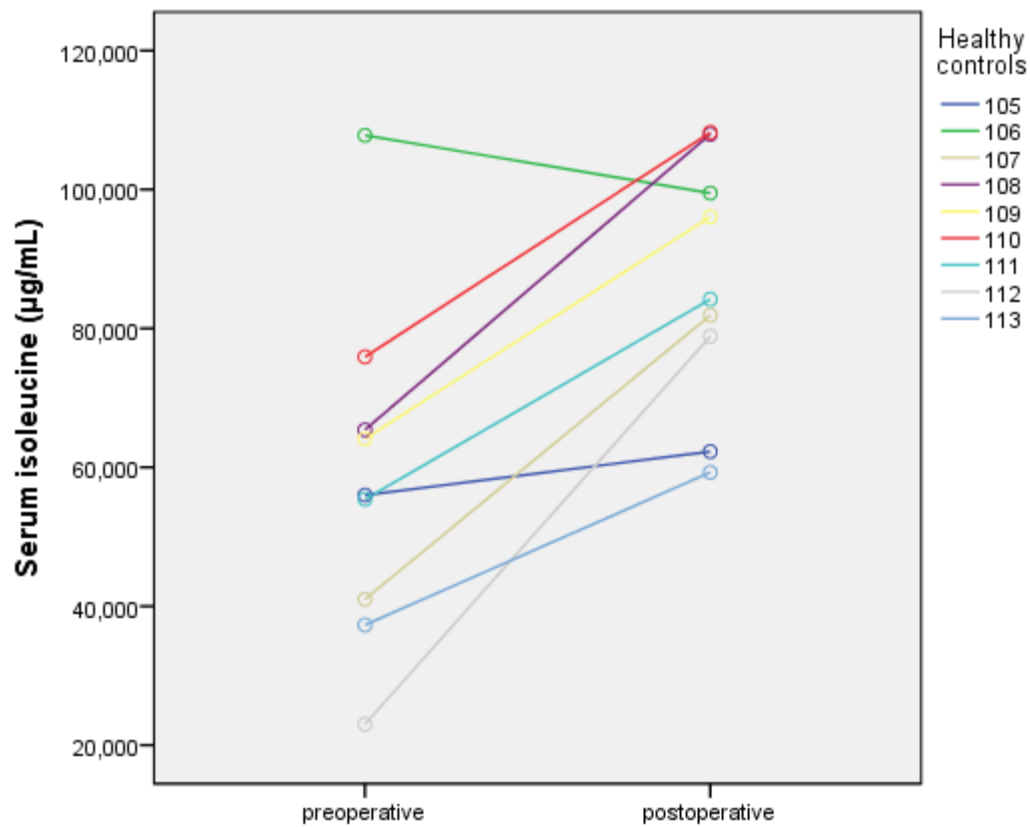

**Lb**

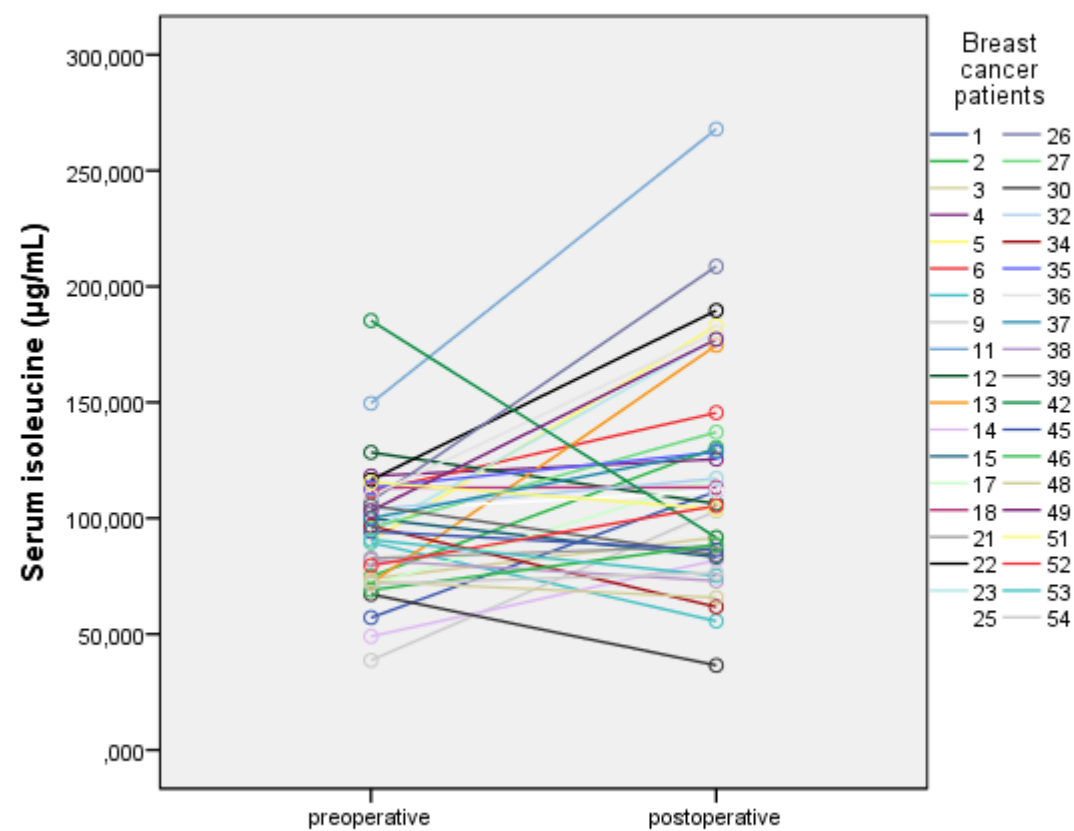

**Ma**

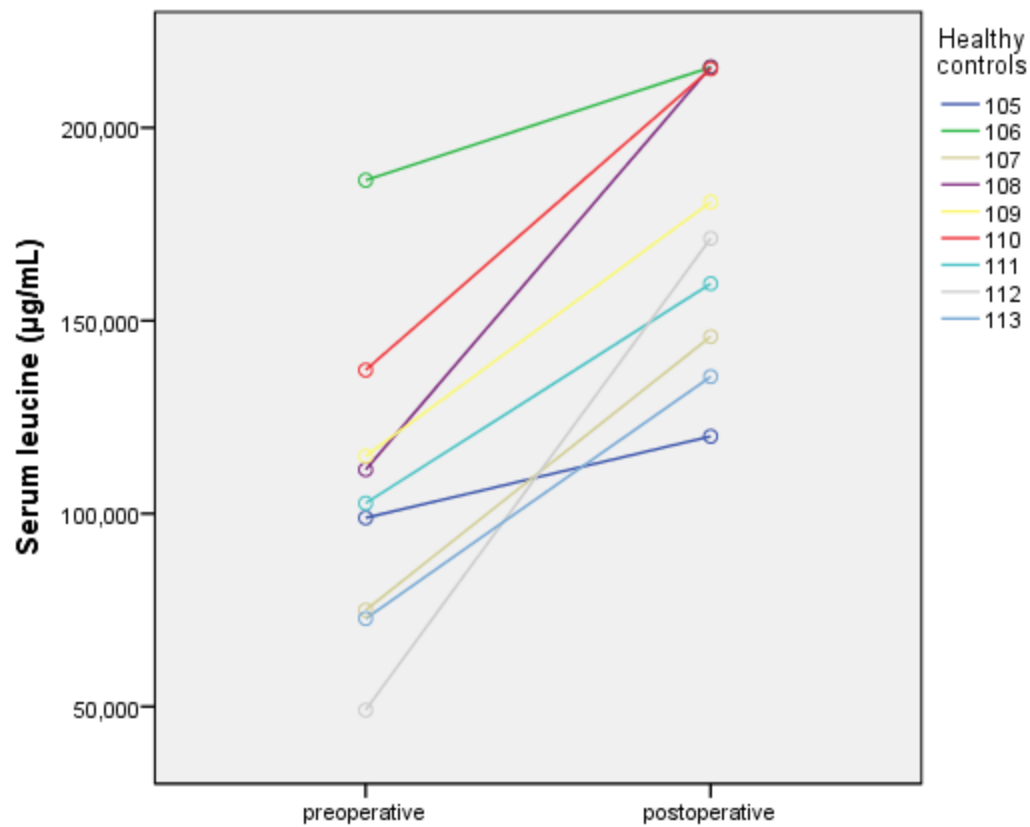

**Mb**

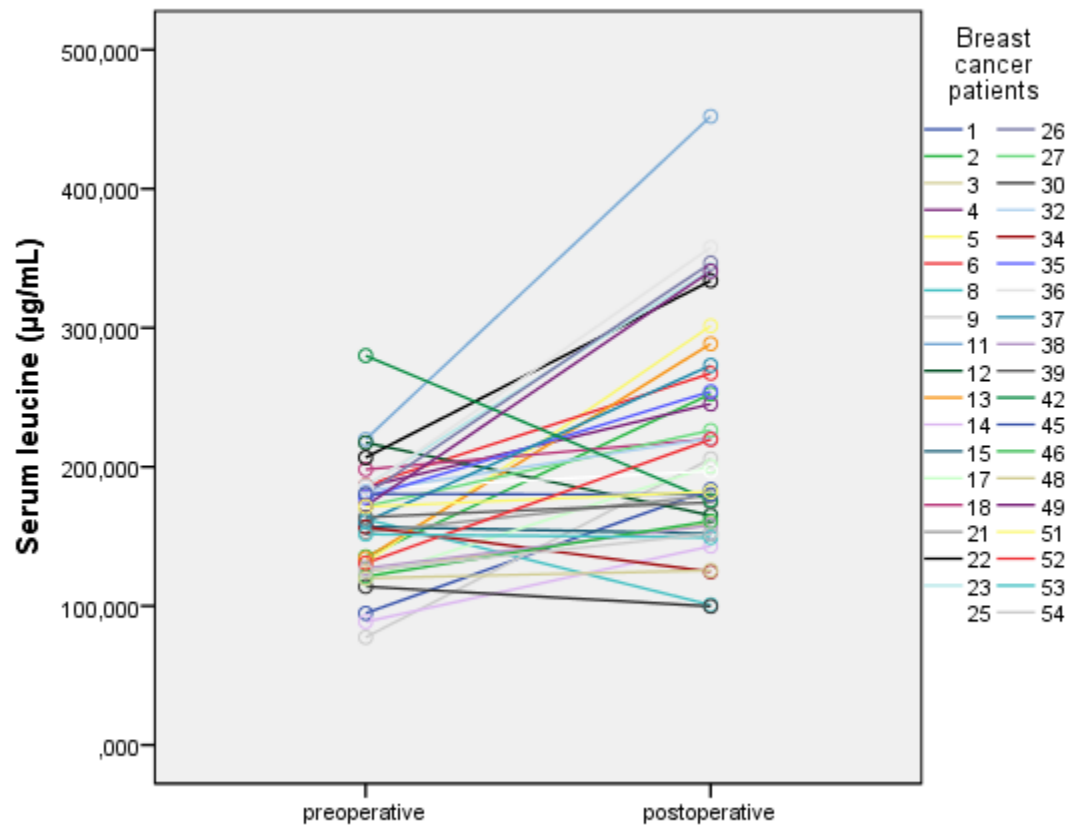

**Na**

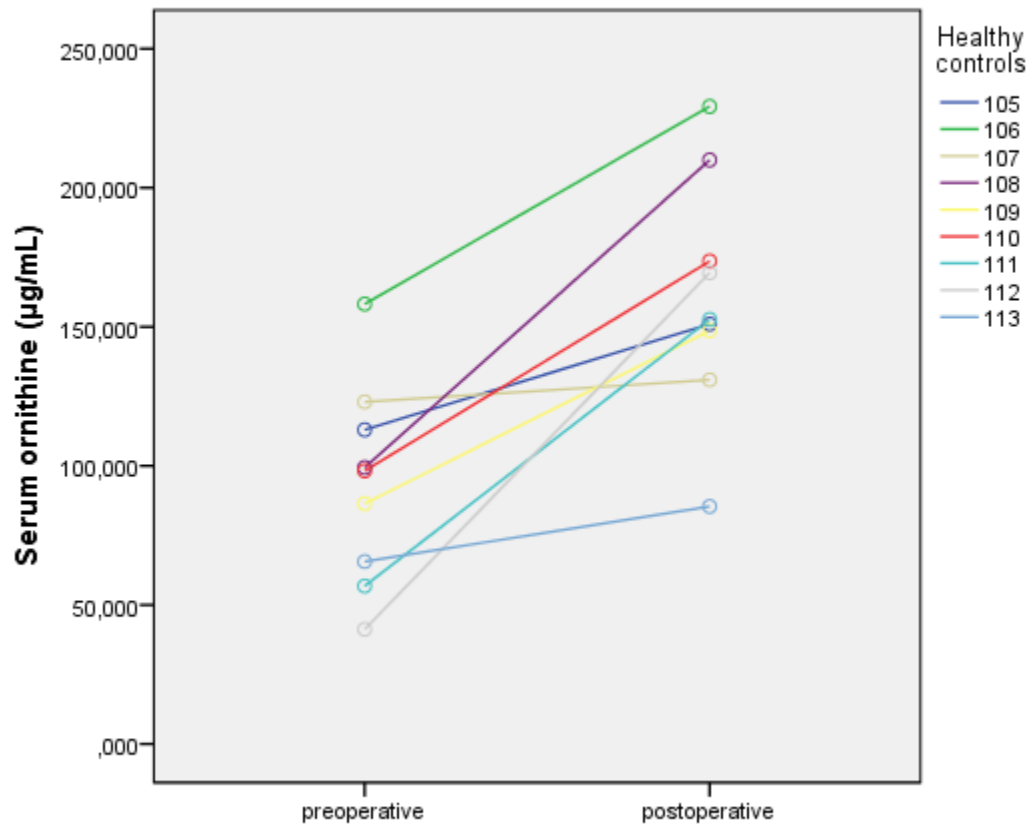

**Nb**

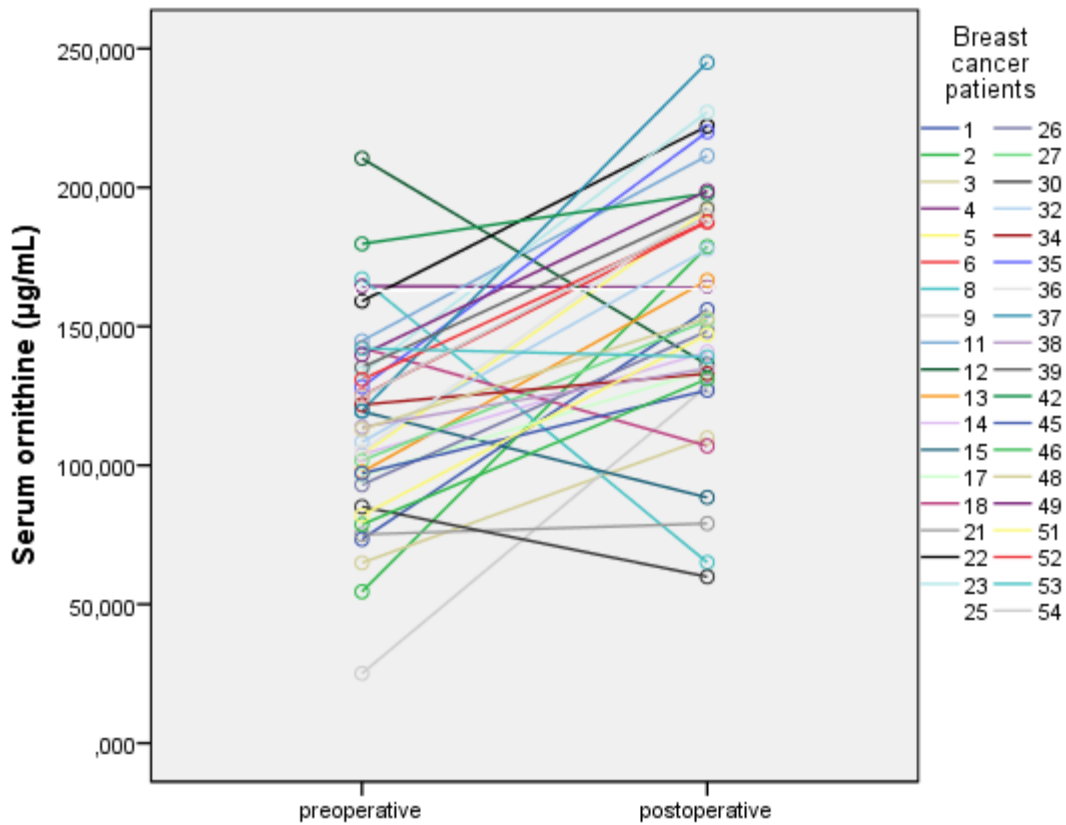

Oa

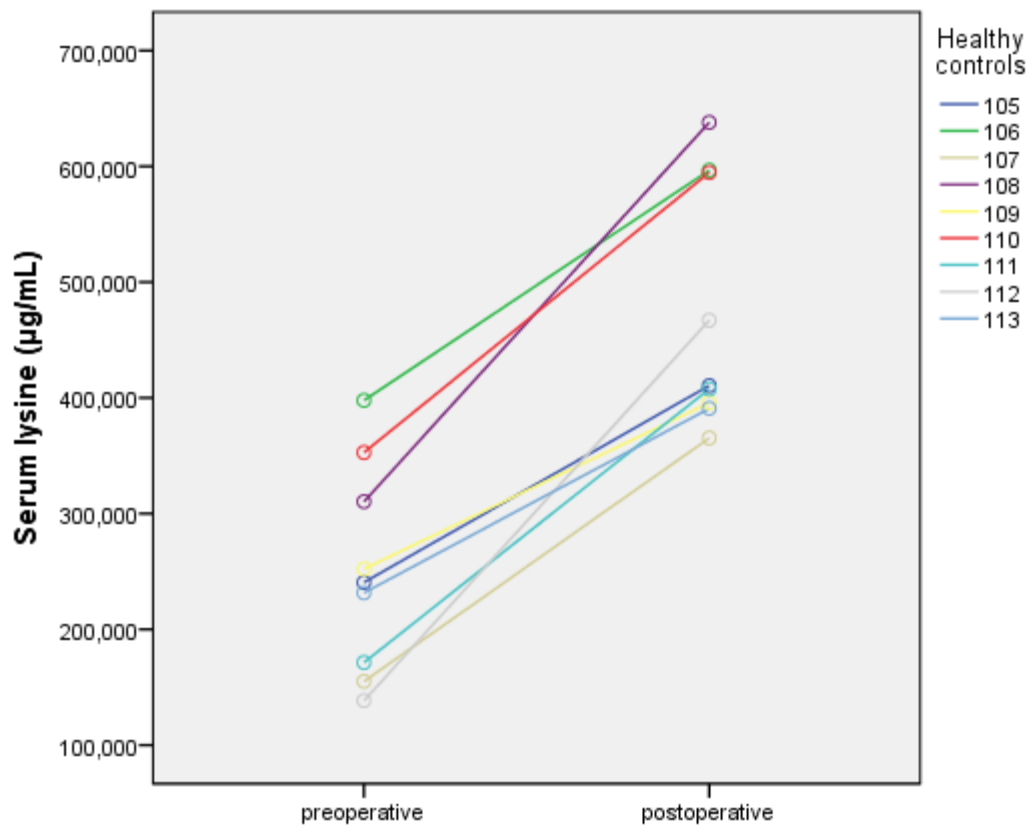

Ob

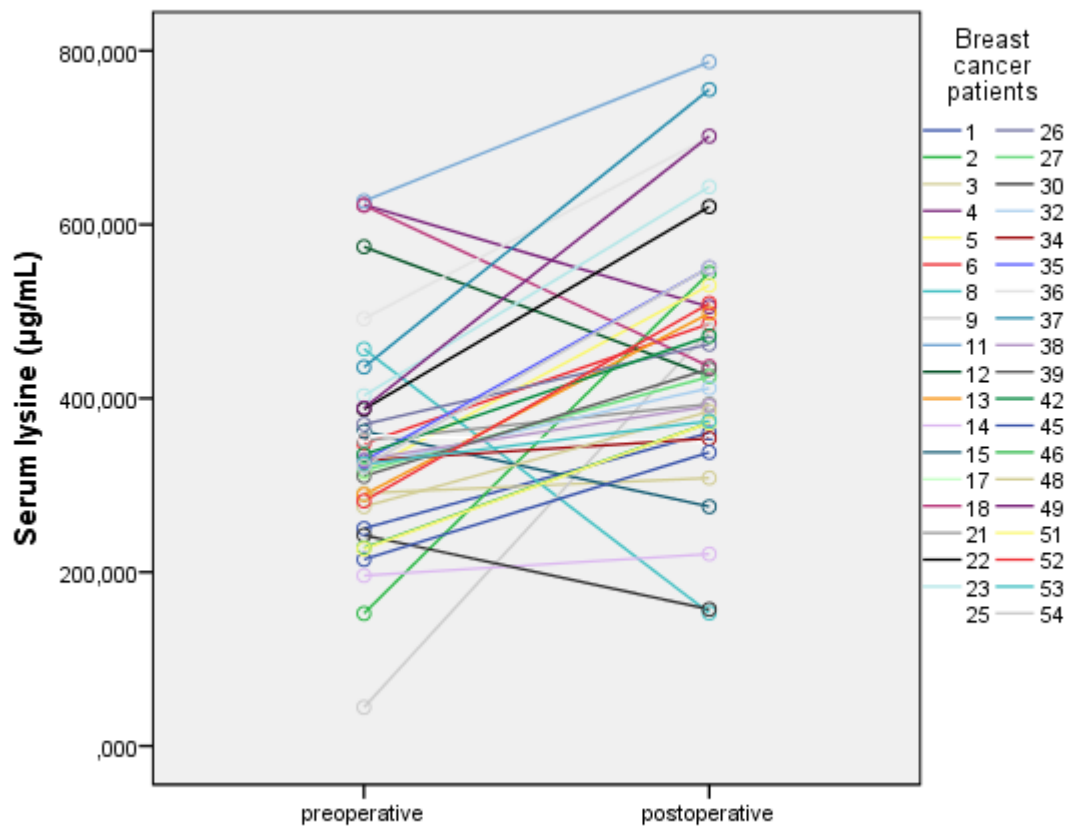

Supplement: Additional file 2: Figure S1 — Raw data analysis of all 15 serum amino acids (A-O) in individual healthy controls (a) and breast cancer patients (b) before and after surgery. [file 1479-5876-11-290-S2.pdf]
